# Molecular Networks of Human Muscle Adaptation to Exercise and Age

**DOI:** 10.1371/journal.pgen.1003389

**Published:** 2013-03-21

**Authors:** Bethan E. Phillips, John P. Williams, Thomas Gustafsson, Claude Bouchard, Tuomo Rankinen, Steen Knudsen, Kenneth Smith, James A. Timmons, Philip J. Atherton

**Affiliations:** 1 Graduate Entry Medicine and Health, Royal Derby Hospital, Derbyshire, United Kingdom; 2 Karolinska Institutet, Stockholm, Sweden; 3 Pennington Biomedical Research Center, Baton Rouge, Louisiana, United States of America; 4 Medical Prognosis Institute, Hørsholm, Denmark; 5 XRgenomics Ltd., Leicestershire, United Kingdom; 6 Loughborough University, Loughborough, United Kingdom; Georgia Institute of Technology, United States of America

## Abstract

Physical activity and molecular ageing presumably interact to precipitate musculoskeletal decline in humans with age. Herein, we have delineated molecular networks for these two major components of sarcopenic risk using multiple independent clinical cohorts. We generated genome-wide transcript profiles from individuals (n = 44) who then undertook 20 weeks of supervised resistance-exercise training (RET). Expectedly, our subjects exhibited a marked range of hypertrophic responses (3% to +28%), and when applying Ingenuity Pathway Analysis (IPA) up-stream analysis to ∼580 genes that co-varied with gain in lean mass, we identified rapamycin (mTOR) signaling associating with growth (*P* = 1.4×10^−30^). Paradoxically, those displaying most hypertrophy exhibited an *inhibited* mTOR activation signature, including the striking down-regulation of 70 rRNAs. Differential analysis found networks mimicking developmental processes (*activated* all-trans-retinoic acid (ATRA, Z-score = 4.5; *P* = 6×10^−13^) and *inhibited* aryl-hydrocarbon receptor signaling (AhR, Z-score = −2.3; *P* = 3×10^−7^)) with RET. Intriguingly, as ATRA and AhR gene-sets were also a feature of endurance exercise training (EET), they appear to represent “generic” physical activity responsive gene-networks. For age, we found that *differential* gene-expression methods do not produce *consistent* molecular differences between young versus old individuals. Instead, utilizing two independent cohorts (n = 45 and n = 52), with a continuum of subject ages (18–78 y), the first reproducible set of age-related transcripts in human muscle was identified. This analysis identified ∼500 genes highly enriched in post-transcriptional processes (*P* = 1×10^−6^) and with negligible links to the aforementioned generic exercise regulated gene-sets and some overlap with ribosomal genes. The RNA signatures from multiple compounds all targeting serotonin, DNA topoisomerase antagonism, and RXR activation were significantly related to the muscle age-related genes. Finally, a number of specific chromosomal loci, including 1q12 and 13q21, contributed by more than chance to the age-related gene list (P = 0.01–0.005), implying possible epigenetic events. We conclude that human muscle age-related molecular processes appear distinct from the processes regulated by those of physical activity.

## Author Summary

A fundamental challenge for modern medicine is to generate new strategies to cope with the rising proportion of older people within society, as unaddressed it will make many health care systems financially unviable. Ageing impacts both quality of life and longevity through reduced musculoskeletal function. What is unknown in humans is whether the decline with age, referred to as “sarcopenia,” represents a molecular ageing process or whether it is primarily driven by alterations in lifestyle, e.g. reduced physical activity and poor nutrition. Because the details of such interactions will be uniquely human, we aimed to produce the first reproducible global molecular profile of human muscle age, one that could be validated across independent clinical cohorts to ensure its general applicability. We combined this analysis with extensive data on the impact of exercise training on human muscle phenotype to then identify the processes predominately associated with age and not environment. We were able to identify unique gene pathways associated with human muscle growth and age and were able to conclude that human muscle age-related molecular processes appear distinct from the processes directly regulated by those of physical activity.

## Introduction

Discovery of the biological determinants of muscle mass and functional molecular phenotypes has substantial bearing on the fields of human performance (e.g. hypertrophy, strength or endurance adaptations [1], [2]) and human health (countering muscle atrophy and deconditioning occurring in older age or with conditions such as cancer cachexia [3], [4], respiratory disease or medically enforced immobilization (e.g. hospitalized bed-rest, cast-immobilization [5], [6])). Resistance exercise (RE) training (RET) is an effective intervention to increase muscle mass in many, but not all people, and thus provides an excellent opportunity to study gene-network regulation during muscle hypertrophy and the proposed relationship to muscle aging. Many exogenous factors may influence RET-induced hypertrophy including manipulation of exercise volume [7], intensity [8] and adequate macronutrient availability [9] all of which interact with an individual's genotype to determine muscle growth. Establishing the molecular diagnostics that enable a personalized approach to tackle ageing has great appeal.

To date, the molecular regulation of muscle hypertrophy has mostly focused on aspects of post-genomic signaling, with early work concluding that canonical insulin-like growth factor (IGF-1) signals control muscle hypertrophy though a phosphatidyl-inositol-3 kinase/protein kinase B/mechanistic target of rapamycin (PI3K/AKT/mTORc1) pathway [10], abbreviated to mTOR. This protein complex can control cell growth through two mechanisms; firstly, mTORC1 regulates *efficiency* of translation through inducing phosphorylation of its substrates, ribosomal protein S6 kinase (S6K1) and 4E binding protein 1 (4EBP1) and, secondly, mTORC1 increases translational *capacity* through regulating ribosomal RNA (rRNA) production within the nucleolus [11]. There are however conflicting data regarding the importance of mTOR regulation (protein phosphorylation or target gene mRNA responses) or its up-stream regulators, and acute anabolic or chronic growth responses to resistance exercise [12]–[17] reported from the same laboratories, indicating that important biological rather than methodological issues remain to be identified.

More recent evidence indicates that the mechanisms regulating muscle hypertrophy go beyond the canonical IGF-1/PI3K/AKT/mTORc1 pathway. While circulating IGF-1 concentrations do not determine RET-induced hypertrophy in humans [18], hypertrophy has now been shown to potentially occur through both PI3K-AKT [19] and mTOR [20] *independent* pathways, even in pre-clinical models. Perhaps the most convincing observation in favor of a more divergent regulation of muscle growth, is the fact that disparate exercise modes (e.g. RET vs. endurance exercise training (EET)) can produce similar protein signaling patterns in humans [21]. This suggests that the molecules, so far studied, are pleiotropic and in our opinion probably important for *any* type of tissue remodeling, regardless of the final physiological phenotype [22]. Therefore, a more innovative approach is needed to define links between molecules and ensuing *in vivo* physiological adaptations, than can be achieved with targeted western-based molecular profiling.

Exercise training has also been postulated as a key tool to reverse the impact of ageing on human skeletal muscle phenotypes [23], [24]. Yet, while some ‘genomic features’ of ageing have been reported [25], we have noticed that the available global molecular profiles of human muscle [23], [24], [26], [27] do not identify consistent molecular features. Furthermore, our recent work has highlighted that physiological adaptations to exercise, whether that be hypertrophy [28] or aerobic function [29], are highly heterogeneous in humans, implying that exercise may not be able to “reverse” muscle ageing [23] for some people. For example, following >10-wk of supervised EET, ∼20% of subjects show no improvement in aerobic capacity while ∼30% demonstrate no improvement in insulin sensitivity [30], [31]. Similarly, we reported muscle hypertrophy ranging from 0.8 to 6.0 kg [28], while Raue *et al* reported changes in muscle cross-sectional area (CSA) ranging −1.2 to +10.4 cm^2^ [24]. Both of these RET studies reported that the outcome of supervised progressive RET did not relate to pre-existing differences in characteristics (i.e., gender, age, pre-existing muscle mass, physical activity levels or dietary habits) indicating that there is not a simple explanation for the heterogeneity of the gains in lean mass [28], [32].

In recent years, we have focused on using the heterogeneous responses to exercise training and OMIC techniques to uncover molecular networks regulated by EET [33] [29] or generate molecular predictors of trainability [34], directly in humans. The aim of the present work is to produce the first reproducible molecular signature of human muscle age, and examine how such a profile relates to new and established exercise adaptation gene networks. We generated new gene-chip profiles from muscle samples derived from two independent clinical cohorts, with a continuous range of ages (18–79 y) and which originate from distinct environments (UK and USA) and which were independently processed in the laboratory. We also generated a new data set of paired global RNA-responses to a supervised 20-wk RET program (N = 44), as well as utilizing various sets of published acute-RET and chronic-EET gene-chips (total N = 200) data sets. Finally, Ingenuity's new IPA up-stream analysis tool [35] was used to identify key features, within these novel age and exercise signatures, to provide independent and robust molecular insight into the heterogeneous nature of muscle hypertrophy, and human muscle age.

## Results

**Figure 1:**
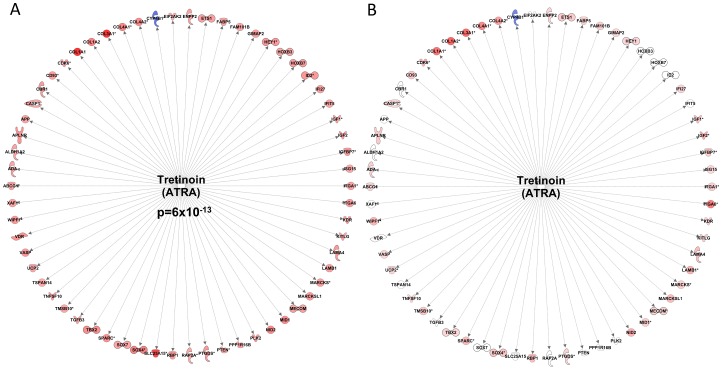
Resistance exercise training induces an all-trans retinoic acid differential gene expression signature that is common with endurance exercise training. A) Forty-four subjects completed a 20 wk supervised resistance exercise training program (RET) and biopsy RNA was profiled before and 72 hr after training. Following SAM analysis of the 38 subjects that demonstrated a clear physiological gain, the gene list was uploaded to the Ingenuity Pathway Analysis database (IPA) and the up-stream regulators were identified using IPA's new up-stream tool. An All-trans-retinoic acid (Tretinoin) gene expression literature network was found to have a significant overlap with the RET dataset (p = 6×10^−13^). Furthermore, the direction changes of the common transcripts were sufficiently similar enough to conclude that ATRA like activity was increased (Z-score = 4.5). B) We re-evaluated our earlier RNA responses to endurance exercise training (EET) study which involved biopsy profiles before and after 6 weeks endurance training in 24 subjects [29], using the IPA tool and while the ATRA was not independently significant the vast majority of the genes within the RET ATRA signature were regulated in an identical manner by EET.

The paired differential analysis, comparing expression in 38 from 44 subjects before and after RET yielded a dataset of ∼700 regulated genes (Dataset S1) and this gene-list related to a few upstream regulators in IPA (Dataset S2). For logical reasons we used only the 38 subjects that demonstrated a training effect [31]. This list included a 62-gene network (Figure 1A), representing the transcriptional action of all-trans-retinoic acid (Tretinoin) and this signature was highly ‘activated’ following 20-wk RET (Z-score = 4.5 for directional consistency; P-value for transcript overlap (p = 6×10^−13^)). In contrast, aryl hydrocarbon (AhR) and V-Ki-ras2 Kirsten rat sarcoma viral oncogene homolog (KRAS) signaling (Figure S1A) was inhibited, such that repression of connected genes was removed, and thus target mRNAs up-regulated.

We utilised the differential RNA response to 6-wks of EET [29] to address the question of specificity of this RET molecular profile to conditions of muscle growth. We have previously speculated that a core set of gene-networks will be common to all types of exercise and could represent basic determinants of tissue adaptability [22]. Indeed, we found that several significant upstream transcriptional regulators from the RET profile were also significant significantly regulated by EET (Dataset S1) and the majority of individual genes in the RET RNA signatures were activated in an identical manner between RET and EET (Figure 1B and Figure S1B). Thus while differential expression in the Derby Resistance Exercise Training (DRET) study clearly identifies a number of tissue remodeling related processes, these are not specific to exercise training modality.

We then utilized the dataset from the Trappe laboratory (GSE28422, ‘Study A’ from GEO). We removed the following samples (or the related paired sample) because the following samples appeared to have technical issues; GSM702424, GSM702439, GSM702431, GSM702421, GSM702414, GSM702436 and GSM702405 GSM702393, GSM702399, GSM702411. Unfortunately we were declined access to the phenotype data (individual muscle growth changes) for each subject (Dr S Trappe, personal communication) and thus we were unable to re-analyze their data to capture the growth related responses, as their data-set included a number of ‘non-responders’ for lean mass gains. Nonetheless, we used the acute RE response dataset found in the Trappe study (GSE28422) so that we could explore the overlap between the acute RNA responses and chronic RET adaptation. What we noted was that <2% of the RNA changes that occur with chronic adaptation in the DRET study, were regulated in response to acute resistance exercise.

### Molecular networks associated with muscle hypertrophy

Of greater physiological importance was our effort to identify genes which link to the *magnitude* of muscle hypertrophy in humans. Quantitative SAM analysis [3], [36] identified 642 probe-sets and interestingly the majority of genes identified were negatively correlated with gains in lean mass (Dataset S2). The probe-set list was imported into IPA (filtered at a 5% FDR) and 384 genes could be mapped to the IPA database for pathway analysis. The enrichment score generated for the EIF2 canonical pathway enrichment was extremely significant (*P*-value = 1×10**^−64^**) and the combination of these observations indicates that the gene-list was both predictable (valid with extensive literature) and statistically very robust (Figure S2). We identified a number of regulators that could be responsible for regulating the transcriptional signature that correlated to gain in lean mass (Dataset S2).

**Figure 2:**
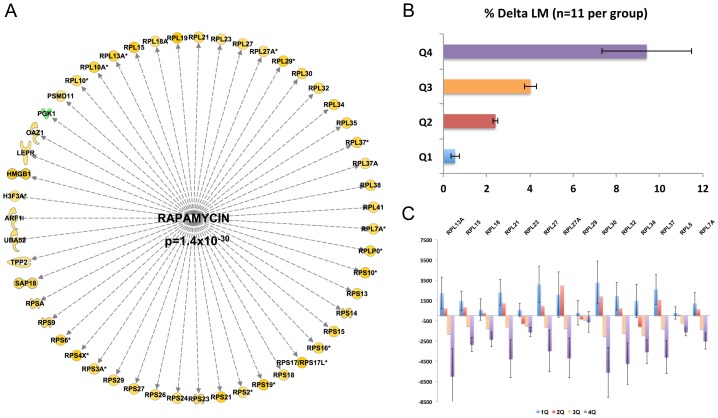
Inhibition of the mTOR-related expression network is correlated with gains in lean mass following RET. A) Quantitative SAM analysis was used to relate the change in RNA expression in response to 10 wk RET in 44 subjects. The change in gene expression was related to the change in lean mass (%) and a false discovery rate calculated based on permutation of the subject labels. Data were imported into IPA and 384 genes (FDR<5%) could be mapped to the data-base for up-stream analysis. An active rapamycin signature, equating to *inhibition* of mTOR signaling was discovered (Z-score = 2.8 for directional consistency; *P*-value for transcript overlap p = 1.4×10^−30^). B) Given the strength of the negative statistical association between the rapamycin signature, we then plotted the data to establish the precise nature of the relationship. We presented the mean gains in lean mass by quartiles establishing that 25% of the subject demonstrated negligible changes in lean mass. C) We selected a representative subset of the genes from Figure 2A and plotted the mean changes with respect to lean mass changes. This established that those with the greatest lean mass actually had a *reduction* in mTOR related genes with RET and not simple a lesser increase as one might have expected from first inspection of Figure 2A.

Figure 2A presents the most striking finding, an active rapamycin signature, equating to *inhibition* of mTOR signaling [37]. This signature was comprised of genes that almost entirely *negatively* correlated with lean-mass gain (Z-score = 2.8 for directional consistency; *P*-value for transcript overlap p = 1.4×10**^−30^**). In short, subjects that demonstrated the largest gains in lean tissue mass following 20-wks of RET have suppressed mTOR signaling over the training period, a novel and controversial observation (all raw data was manually checked for consistency of direction). A second major transcriptional regulator was MYC, and the gene-list driving its inclusion (Z-score −5.8 for directional consistency; p-value for transcript overlap p = 4×10^−43^) overlapped with the rapamycin network, equating to the inhibition of MYC activity. MYC can be an upstream activator of mTOR signaling in cell culture systems [38]. Thus these two robust observations are consistent, and notably the signature evidence is based on entirely independent data.

Close examination of the rapamycin associated gene network (Figure 2A) reveals ∼30 ribosomal RNA genes (a total of 70 genes were in the lean mass gain associated list but not all featured in the data-base of rapamycin regulated genes). The remaining genes (Figure 2A) belonged to metabolic processes and other facets of protein metabolism or signaling. To more easily appreciate the characteristics of those subjects that were found to have the greatest increase in lean tissue mass combined with a reduction in ribosomal gene abundance, we plotted the quartile response in lean mass (Figure 2B). Baseline lean mass could not explain our observation and in fact the four groups had the same mean age, physiological characteristics, while the highest and lowest quartiles for lean mass gain had exactly the same proportion of males and females (Table 1). Figure 2C specifically illustrates a subset of the ribosomal RNA genes, however all other rRNA genes were consistent with this plot. There was an almost universal pattern of down-regulation observed in high responders (shown in purple) while subjects that were unable to increase muscle mass (shown in blue) substantially up-regulated these genes, as would be expected from the qSAM statistical analysis (FDR<5%) in Figure 1A.

**Table 1:** Baseline biochemical and physiological characteristics of the DRET cohort.

Phenotype	Q1 (n = 11)	SE	Q2 (n = 11)	SE	Q3 (n = 11)	SE	Q4 (n = 11)	SE
**Basal upper lean leg mass**	5550	±442	3284	±438	3595	±380	4689	±239
**Change in lean mass (%)**	0.5	±0.2	2.4	±0.1	4.0	±0.3	9.4	±2.1
**Age (yr)**	49.2	±5.5	47.5	±4.6	53.1	±5.7	51.1	±5.7
**Male∶ female ratio**	8∶3	-	4∶6	-	4∶6	-	8∶3	-
**Pre-training BMI**	25.5	±0.9	25.9	±0.6	25.9	±0.8	26.6	±1.2
**Pre-training fasting Insulin**	3.6	±0.7	5.0	±0.6	5.0	±1.0	4.2	±0.6
**Pre-training fasting Glucose (mmol/l)**	5.4	±0.2	5.7	±0.1	5.5	±0.3	5.7	±0.2
**Type I fibres**	34.8	±0.7	32.9	±0.8	31.4	±0.7	31.7	±1.1
**Type IIA fibres**	31.0	±0.6	32.0	±0.6	34.1	±0.6	33.3	±0.8
**Type IIX fibres**	33.9	±0.8	34.7	±1.0	33.8	±0.7	34.9	±1.1
**Leg strength (Newtons)**	2270	±264	2206	±265	2073	±166	2149	±124

**Figure 3:**
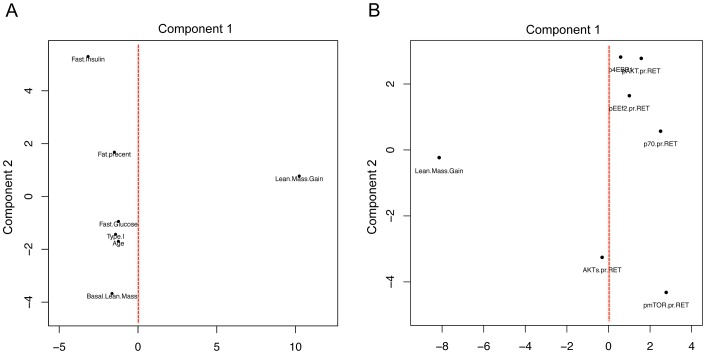
Using principal component analysis to evaluate the relationship between physiological and acute protein signaling events to RET induced gains in lean mass. A) Change in lean mass following 20 wk RET and a number of physiological parameters which demonstrated the most variance were scaled to a common value and plotted using principal component analysis in R. Principal component (PC) 1 captured the major variance in lean mass gains across subjects however none of the commonly postulated physiological parameters varied with lean mass (linear regression analysis demonstrated no significant association also). PC2, the second largest proportion of independent variance also demonstrated no association between factors such as fiber type or age and gains in lean mass. B) Phospho-protein signaling 2 hr after a combined exercise and nutrition acute intervention (to promote anabolic signaling) were scaled and plotted with change in lean mass following 20 wk RET. Again these acute signaling events shared little common variance in either PC1 or PC2 with changes in lean mass with 20 wk RET.

Thus, our analysis strategy enabled the discovery of an entirely novel *in vivo* feature of the mTOR growth pathway, while standard differential RNA expression analysis (pre vs. post sample) could not. We also plotted the relationship between physiological characteristics, protein-phosphorylation during acute net anabolic situations (resistance exercise coupled with feeding) with lean mass gain in these subjects using principal component analysis (PCA). Selected variables were scaled and principal components 1 and 2 are presented. In Figure 3A, it is abundantly clear that none of the metabolic or physiological characteristics shared variance with the main component capturing lean mass gain variation following RET. Likewise, while protein kinase abundance or protein kinase activation status varied in a manner consistent with the literature, none of these acute net anabolic responses were correlated to gains in RET induced lean mass or shared variance with gains in lean mass when studied prior to 20-wk RET (Figure 3B). In short, only the *unbiased* transcriptomics method was able to identify a biological profile distinguishing between high and low responders for lean mass gain.

### Age muscle gene networks

Identification of the determinants of skeletal muscle mass has obvious implications for treating age-related sarcopenia. There is no longitudinal molecular analysis of ageing muscle in humans. However using cross-sectional gene-chip data-sets, attempts have been made to identify age-related gene expression changes. For example, Melov *et al.*, reported that differences in gene expression between a cohort of young and old subjects can be removed through a RET program [23]. RET removed some aspect of the ‘inactivity’ related component of the difference between young and old subjects but when we contrasted their age-signature with other publically available muscle age datasets [23], [24], [26], [27] no overlap was apparent.

**Figure 4:**
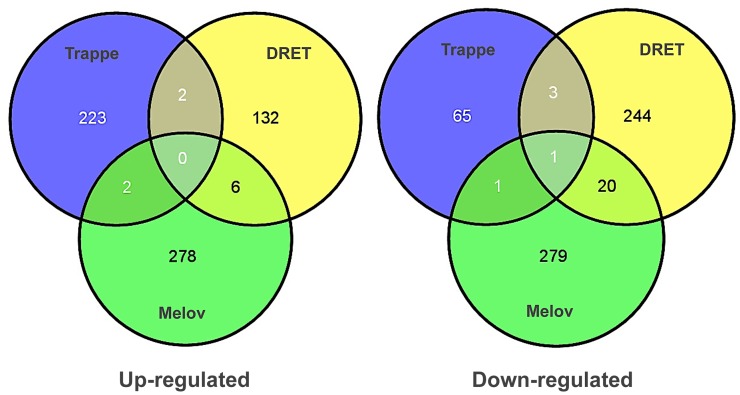
Differential gene expression analysis, contrasting young and old subjects, does not produce a reliable biomarker signature of age. Several attempts have been to define a set of genes that differ in skeletal muscle between young and old human subjects. We re-analysed three of the most robust and largest human studies with common methods, including our new study, and contrasted the genes identified to be differentially regulated using SAM analysis and Gene Ontology analysis. No common pattern of differential gene expression could be found using this analysis method indicating that no prior gene signature for muscle ageing can be considered a reliable marker of muscle age in humans. Gene ontology analysis found that both the Trappe and Melov data sets had modest enrichment in mitochondrial genes, which were down-regulated with age however this was not true for the DRET study and both Melov and Trappe data-sets had elderly with much lower physical fitness levels making it impossible to attribute these changes to age *per se* with differential expression analysis.

To investigate this issue further, we utilised the Melov *et al.*, data and the data from Trappe and our lab. We used SAM analysis to compare young with old subjects in each study. For the Trappe study we had 13 young (20–30 y) and 11 old subjects (>80 y). We used baseline samples from DRET, 10 young (20–29 y) versus 16 old subjects (64–75 y), and finally we re-analysed the Melov *et al.*, data [23] which had 26 younger (18–28 y) and 25 older subjects (65–85 y). As can be clearly observed in Figure 4 there was no overlap between the three studies indicating that a reproducible set of ‘ageing’ genes cannot be generated with this statistical or experimental approach. Our re-analysis of the GO analysis (using DAVID) of earlier studies [26], [39], using the appropriate back-ground files [40], also confirmed that mitochondrial RNA changes in ageing cannot be claimed as being a reproducible hallmark of ageing, despite the presumed association with inactivity. Re-analysis of the Melov *et al.*, data did identify a mitochondrial gene expression signature (less significant than the original analysis due to comparison with a more appropriate ontological background) but in that study the older subjects were substantially de-trained and aerobic function was not presented. This gene-set is also known to reflect physical activity [29] and inactivity in humans [41] and thus it shouldn't be attributed to age *per se* anyway. To consolidate the conclusion that there was no common finding across these three studies, we entered the individual gene-lists in a gene ontology analysis to evaluate if some common pathways were enriched in each list, even if the member genes differed. Only 1 ontological group was common to 2 from the 3-gene lists and it related to mitochondrial processes indicating that even when the older subjects have a lower physical capacity a decline in mitochondrial genes is not always a prominent feature of age-related changes.

Therefore, an alternative approach to identify age-related gene expression profiles in human muscle was required. To achieve this we utilised QSAM, which we have previously applied and validated to some extent for human studies [3]. We applied QSAM to generate a list of muscle transcript levels, which correlated with subject age (20–75 yr), with correction for multiple testing. This allowed us to identify genes that either negatively or positively associated with the subject age. This has not been attempted before because previous studies did not have a sufficiently wide and continuous range of ages to generate such data [23], [24], [26], [39]. However, such an analysis would be of limited value if some of the observations could not be independently reproduced, using a distinct set of clinical samples. To this end, we generated 52 new U133+2 profiles (17–63 yr) from muscle biopsy samples from the HERITAGE Family Study [42] and were able to identify a set of 580 genes or transcripts (Dataset 3) which were correlated with age in both clinical studies and which was enriched in post-transcriptional and chronic disease traits (Figure S3) but not mitochondrial related gene-sets.

**Figure 5:**
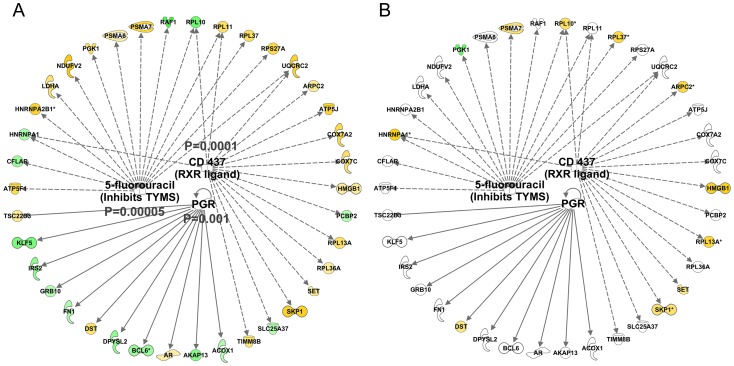
Quantitative SAM analysis using a continuum of age versus gene expression produces network hubs that are activated with human muscle age. Using a total of 97 U133+2 Affymetrix gene-chips newly produced from two independent studies, the DRET study and the HERITAGE family study we produced a novel analysis that relied on the full age-range present in these data sets. A) We first found a set of genes that co-varied with age in the DRET study and then confirmed that 580 of these were also related to age in the HERITAGE study. Mitochondrial genes were not a feature of this linear age vs gene analysis. We then mapped the Affymetrix probe-sets to the IPA database and examined the up-stream analysis output. We found in IPA that the age-related dataset was consistent with the activation of the PGR (z-score = 2.6 and p-value = 0.001) and RXR (z-score = 2.0 and p-value = 0.0001) proteins and 5-fluorouracil agonism (Z-score = 2.2 and p-value = 0.0005). B) We noted that some members of these age-related networks were also associated with lean mass gains in humans. However about 50% of the common genes were positively associated with lean mass gain and age; and 50% were regulated in a discordant manner. Clearly some responses can be causal, some may be purely correlative and some may represent compensatory events.

We found in IPA that the age-related dataset was consistent with the activation of the PGR (z-score = 2.6 and P-value = 0.001) and RXR (z-score = 2.0 and p-value = 0.0001) proteins and 5-fluorouracil agonism (Z-score = 2.2 and P-value = 0.0005, Figure 5A). Each mediator orchestrated a set of either positive (yellow) or negatively (green) age-correlated genes such that both overlap and direction were similar to the literature-constructed networks. Critically, these networks were not significantly related to the lean-mass associated gene-list (Figure 5B) or differentially regulated by either acute RE (Figure S4A) or chronic endurance exercise (Figure S4B). Thus it is unlikely that these new age-related observations reflect simple confounding factors, such as exercise training or being physically active.

**Figure 6:**
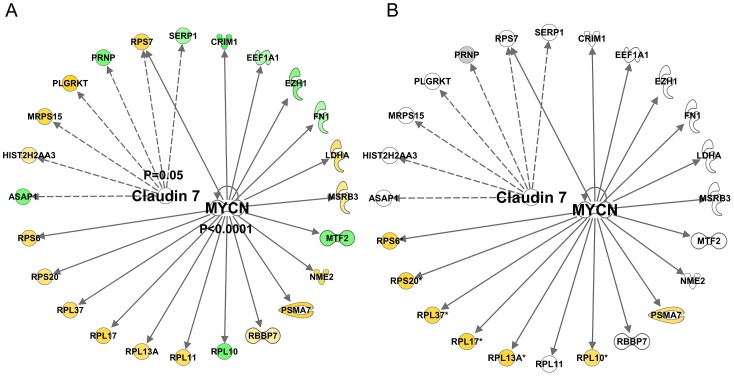
Quantitative SAM analysis using a continuum of age versus gene expression produces network hubs that are inhibited with human muscle age. Using a total of 97 U133+2 Affymetrix gene-chips newly produced from two independent studies, the DRET study and the HERITAGE family study we produced a novel analysis that relied on the full age-range present in these data sets. A) We first found a set of genes that co-varied with age in the DRET study and then confirmed that 580 of these were also related to age in the HERITAGE study. Mitochondrial genes were not a feature of this linear age vs gene analysis. We then mapped the Affymetrix probe-sets to the IPA database and examined the up-stream analysis output. We found in IPA that the age-related dataset was consistent with including c-MYC (z-score = −2.8 and p-value<0.0001) and CLDN7 (z-score = −2.6 and p-value = 0.05). B) A few members of these age-related networks were also associated with lean mass gains in humans and this included mTOR regulated genes, which were negatively associated with increasing age and thus in contrast to the lean-mass association.

**Figure 7:**
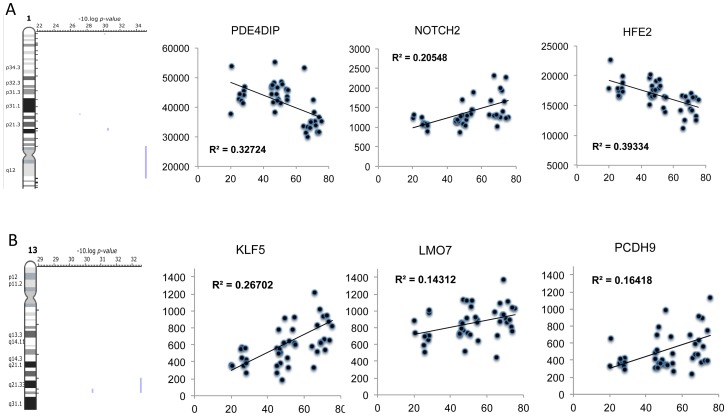
Positional enrichment analysis identified age-related genes that were over-represented on 4 chromosomes including Chromosome 1 and 13. A plot of selected genes found to be over-represented at 1q12 and 13q21. A) Positional enrichment analysis identified 3 genes at each loci and B) the relationship with age of each gene was plotted with the linear correlation coefficient provided and the FDR derived from the qSAM analysis (FDR<5%). Genes at these loci included proteins that are known to influence mTOR related signalling.

There was also *inhibition* of certain protein mediators with age (Figure 6A) including c-MYC (z-score = −2.8 and p-value<0.0001) and CLDN7 (z-score = −2.6 and p-value = 0.05). Again, no clear relationship with acute exercise or endurance training was apparent (Figure S5), while a closer association with genes related to gains in lean mass was noted (Figure 6B) with some key exceptions. Notably inhibition of MYC was predicted in *both* the lean-mass and age-related gene lists (with gene-correlations in the same direction) which we would not expect if muscle ageing was simply the ‘opposite’ of muscle growth or lack of response to exercise training. Furthermore, large differences in gene expression still existed when comparing the age groups and the pre and post-training samples in the Trappe dataset (data not shown). The age-related expression signature was also related to RNA signatures in the Broad Connectivity database, including multiple serotonin antagonists and appears opposite to DNA topoisomerase inhibition (Dataset S4). Finally, we examined whether the age-related genes were over represented at genomic loci using Positional enrichment analysis [43]. Both Chromosome 1 (q12) and 13 (q21.33) had significant hits and the genes associated with those locations can be found in Figure 7A and 7B and the remaining analysis in Dataset S5.

## Discussion

We have carried out unbiased molecular analyses on both new and pre-existing human muscle data-sets (acute and chronic exercise, RET or EET and ageing) from which we have been able to define: (i) a ‘generic’ set of molecular networks that are activated irrespective of exercise-training mode in humans, i.e., RET or EET, (ii) the differential effects of acute exercise versus chronic exercise training on molecular networks, (iii) the molecular networks that are *specifically* modulated in relation to the extent of human muscle hypertrophy, and (iv) the first reproducible set of age-related transcriptional changes further supporting our view that large human sample sizes, using unbiased molecular profiling techniques [4], [29], [44] is an important strategy for translational medical science.

### Exercise mode and the specificity of the molecular response

Muscle hypertrophy is the most recognized adaptation to RET. However, there are numerous other adaptations that occur, to support the biochemical, physical and metabolic requirements of a growing muscle. For example, hypertrophy is associated with activation of muscle satellite cells to support growth [45] while RET stimulates angiogenesis proportional to muscle fiber growth (rather than increasing capillary-to-muscle fiber area as EET [46]). Furthermore, along with accretion of contractile proteins, the extracellular matrix (ECM) undergoes substantial remodeling after RET [47]. Clearly then, successful hypertrophy is the summation of complex intra/extra-muscular cross talk to co-ordinate hypertrophy. As such, we believe that teasing out the molecular networks regulating adaptation *in vivo* requires charting the relationship between the modulation of molecular factors with that of the physiological outcome(s) [29].

Our initial analysis revealed that activation of a Tretionin (all-*trans* retinoic acid (ATRA)) and inactivation of aryl hydrocarbon receptor (AhR) are common molecular responses to training, irrespective of exercise mode i.e., RET or EET. ATRA is the active form of vitamin A, which serves as a ligand for two families of widely expressed nuclear receptors; the retinoic acid receptors (RAR) that bind to ATRA and the retinoid X receptors (RXR) that bind to its stereoisomer, alitretionin (9-cis-RA). Although little work exists on ATRA signalling in skeletal muscle, Halevy *et al.* showed exogenous ATRA promoted myogenic cell differentiation [48] which is allied to the function of ATRA amongst various cell types [49]. Given the post-mitotic properties of myonuclei, this may point to a novel link between ATRA-like signalling and aspects of *in vivo* satellite cell activities induced by exercise training. In support of this idea, we also observed IGF-1 and IGF-2 up-regulation in both RET and EET, and this is thought to play a role in satellite cell activation and differentiation [50], [51] [52]. ATRA-like signalling has also been shown to modulate endothelial cell maturation and angiogenesis in tube formation assays [53] suggesting a role for activation of this network in exercise-induced angiogenesis, while angiogenic factors are also associated with satellite cell activation and differentiation [54]. Indeed, we identified up-regulation of homeobox (HOX) genes (e.g. HOXB3/7), which have roles in vascular remodelling and angiogenesis [55]. Thus this collection of genes is likely contributing to vascular remodelling-induced both by RET [56] and EET [57]. In addition, the turnover of ECM components is activated when smooth muscle cells are exposed to exogenous ATRA, thereby suggesting this pathway is involved in ECM remodelling [58]. Indeed, the (activated) ATRA gene list was highly enriched in collagen genes. Finally, while one cannot rule out that the degree of modulation of ECM (which differed between RET and EET) may influence aerobic adaptation [29], [31] it would appear to be a constituent feature of muscle growth and remodelling *per se*.

The AhR is a ligand-activated transcription factor known to mediate the negative effects of environmental xenobiotic contaminants such as dioxin (i.e., TCDD; 2,3,7,8-tetrachlorodibenzo-*[p]*-dioxin). This receptor belongs to the basic-helix-loop-helix (bHLH)/PAS (Period [Per]-AhR nuclear translocator [Arnt]-Single minded [Sim]) family of heterodimeric transcriptional regulators [59]. There have been a number of studies in which physiological clues have been gathered as to the function of AhR. For example, AhR has shown tumour suppressor effects i.e., when receptor levels are down-regulated by siRNA [60] the G1/S transition of the cell cycle and cell proliferation is promoted, suggesting a growth inhibitory role of the receptor. There also exists links between AhR and angiogenesis. Vascular endothelial growth factor (VEGF), a major growth factor that regulates angiogenesis is transcriptionally regulated by hypoxia-inducible factor-1 alpha (HIF-1α) in response to tissue hypoxia and muscle contraction [61]. Hypoxia stabilizes HIF-1α, which forms heterodimers with HIF-1β, also known as the AhR nuclear translocator (ARNT). ARNT can heterodimerize with AhR, minimizing the ability of ARNT to interact with stabilized HIF-1 to induce VEGF production. Finally, matrix remodelling is negatively affected during AhR activation. For example, AhR activation blocks regenerative processes during zebra fish caudal fin regeneration while impairing expression of genes involved in the structure and remodelling of the ECM [62]. Thus, *inactivation* of AhR-signalling may facilitate ECM and vascular remodelling occurring in response to both RET and EET (Figure 1).

The notion that both ATRA and AhR pathways are potent regulators of cell growth, differentiation, ECM remodelling, vascularization, organogenesis and embryogenesis [63] underlines their key roles in tissue development and homeostasis. Moreover, since adaptation to RET and EET both involve aspects of cellular remodelling, muscle satellite cell activities [64], ECM and vascular remodelling, we suggest that ATRA and AhR molecular programs are playing a previously undefined but central role in regulating “generic” features of exercise adaptation [22]. Finally, since ATRA and AhR gene networks that were regulated during long-term exercise training (Figure 1), were not reflective of those modulated in the hours after a single bout of exercise [24], [33], this casts doubt over ascribing formative *purpose* of acute exercise gene networks, which more likely represent stress pathways instigated by unfamiliar activities or simply the acute energy crisis in exercised muscle (in agreement with the lack of a striking ontology profile). This may explain why acute mRNA changes do not overlap with the chronic exercise patterns or, in our hands, relate to the networks that associate with the degree of gain in lean mass (see below).

### Networks activated in a manner directly related to muscle growth

Following the identification of what might be called generic ‘adaptability’ [22] molecular networks, we were interested to see if we could identify any molecular networks that were regulated in *proportion* to the degree of muscle hypertrophy in individual subjects. The justification for this approach was based on the marked heterogeneity in capacity for muscle growth in humans, with gains ranging from 0% to 22% [24], [28], [65]. In the DRET study, we found a similar range of changes in muscle size (−3% to +28%) and the analyses of the gain-related gene networks yielded striking results. We first discovered that there existed a correlation between capacity for human muscle lean mass gains and activity of both c-MYC and mTORc1 sensitive genes, including a large group of ribosomal RNA (rRNA) genes (∼70/560 total rRNAs). We further demonstrated that the nature of the association was not as one would be expecting from pre-clinical research, but rather there was a reduction in rRNA gene expression when greater muscle hypertrophy was observed (Figure 2B).

We speculate that human high-responders to hypertrophy potentially show superior efficiency in protein synthesis (i.e., protein yielded per RNA) and/or a reduction in proteolytic responses to RET. Nonetheless, it should also be noted that while rRNA expression negatively correlated to gain in lean mass, the highest responders *tended* to have higher levels of rRNA pre-training for individual rRNAs and hence the absolute abundance was similar post-training. This pattern of response clearly demonstrates that ‘more’ mTOR activation (e.g. more rRNA production) in humans is neither a *hallmark* nor a *necessity* for gains in lean mass *in vivo*. Our observations in humans also contrast with the molecular responses observed during acute synergist ablation induced hypertrophy [66], raising further doubts over the relevance of such pre-clinical models to inform about physiological muscle growth in humans. Regardless, our data demonstrate that high-responders for muscle hypertrophy evoke an “anti-growth” transcriptional response during a period of successful muscle growth with more studies being required in high or low lean-mass responders to investigate this phenomenon unambiguously.

While the unbiased transcriptional profiling yielded novel insights into human muscle growth responses, we also attempted to link training responsiveness to subject baseline physiology and the acute response of phospho-protein (AKT-mTORc1) signaling in response to an anabolic stimulus (RET combined with optimal nutrition [67]). We used correlation analysis to examine the relationship between key factors that have been speculated to be important for human muscle growth (e.g. body composition, fiber-type, metabolic and signaling molecule status). We found no significant shared variance between any of these variables and gains in lean mass, as presented in Figure 3 using PCA. We utilized PCA for visualizing the integration of physiological and molecular data because it enables an over-view in a single plot of how physiological and molecular aspects may vary within the major ‘units’ of variance of a particular dataset rather than plotting multiple individual scatter plots. By doing this, it becomes easier to visualize when paired relationships are behaving as expected, such as total protein and phospho-protein positions within each principal component. Unusual patterns could be used to identify problem areas such as when detection methods (e.g. antibodies) are poorly functioning.

As can be seen in Figure 3A, while principal component 1 was dominated by the variation in lean mass gains, basal lean mass, fat mass or fasting glucose did not vary. These data are in agreement with previous studies in which baseline physiological status was unable to select out high-responder status to hypertrophy [28]. We integrated the acute changes in phospho-protein signaling under the conditions of RET plus nutrition as an index of growth signaling potential in each subject. This seemed a valid approach as muscle hypertrophy is the product of nutrition and exercise-induced muscle protein synthesis [68]. While pre-training acute “anabolic” signals did not share variance with gains in muscle lean mass (Figure 3A), some weak relationships appeared between acute “anabolic” signaling elements following exposure to RET (Figure 3B) and gains in lean mass. Collectively, this underlines that while acute changes in phosphorylation may associate with those of acute remodeling processes (i.e., muscle protein synthesis responses) they are a poor indicator of future leans mass gains. Previously we have found a degree of dissociation between AKT-mTOR signals [69], [70] and muscle protein synthesis, while others have shown that acute synthesis does not, but some signaling molecules do, relate to future gains in lean mass [71]. We contend that using these individual signals as acute proxies for RET muscle growth is not going to be the most sensitive strategy.

### Age-associated and exercise-associated molecular networks

There is a long established relationship between canonical pathways related to muscle growth and mechanisms that are associated with extended life-span in model organisms [72]. For example, inhibitors of mTORc1 and PI3K (a gene also known as “age-1”) activity can extend life-span in *Caenorhabditis elegans*, *drosophila* and mice [72]. As the number of people living beyond their eighth decade rises, it is expected that skeletal muscle atrophy and dysfunction (sarcopenia and dynapenia respectively) will become an increasing public health challenge [73]. Activation of mTORc1 and muscle IGF-1 signalling is associated with muscle cell growth experimentally while chronic inhibition of mTOR has been predicted to induce muscle frailty in humans [14], [74]. Likewise, reduced S6K1 (ribosomal protein S6 kinase) activity of the multifaceted regulator of cell growth would be thought to impair the retention of muscle mass in humans [75]. Thus there is a clear molecular basis to believe that processes under-pinning ageing, longevity, sarcopenia and muscle growth will be strongly inter-connected.

We found a list of ∼500 genes which *track* with age in human muscle across two independent cohorts, when our analyses utilised a continuum of ages. This contrasts with the irreproducibility of previous RNA versus muscle age datasets. Using this data we then evaluated which age-related gene networks link to muscle growth signalling or a variety of exercise scenarios [24], [29], [56], [76]. The motivation for this is that these factors cannot be independently controlled for in human studies and thus *post hoc* considerations are essential. The first clear observation was that the reproducible age-related gene-list and the lean-mass *gain* related gene-list both had inhibition of MYC as a key transcriptional feature immediately indicating that age and muscle growth are not exact opposites. Further, the key upstream regulators of the age-related gene list (e.g. PGR, RXR, Claudin7) contained a set of genes which were largely unrelated to the RET and EET transcriptomes, or the acute exercise responses [24], [76] for that matter. Claudin-7 activity was inhibited with age (Figure 6) and appears to relate to developmental differentiation and is strongly regulated by androgen signaling and HGF *in vitro* [77]. According to the IPA publication database the RXR ligand, CD437, induces a transcriptional signature that is consistent with age-related gene correlations, involving a network that is for 2/3^rd^ negatively correlated with increasing age and for 1/3^rd^ positively associated with age (i.e. with age KLF5 and IRS2 expression increases). Note that the ATRA signature, characteristic of increased physical activity in our hands, should operate through the RAR pathway. However, there is some overlap in the down-stream gene activation and thus both age-related changes and exercise activate some common features related to Vitamin A biology. In short, it is abundantly clear that the age-related changes in gene expression are not simply the ‘opposite’ [23] of the profiles seen with exercise or exercise training.

Positional enrichment analysis [43] was used to map the ∼500 age-related genes to chromosomal localisation, as attempts to link DNA variants with ageing have so far been only partly successful suggesting that an alternative approach may provide useful insights. We found several loci that yielded a significant enrichment score with ∼1q12 (e.g. PDE4DIP) and ∼13q21 (e.g. LMO7 (FBXO20)) yielding the most significant scores (Figure 7). PDE4DIP is a binding partner of phosphodiesterase 4D (PDE4D) which partners with Rheb to be a cAMP-specific negative regulator of mTORc1 [78]. When PDE4D binds Rheb it inhibits the ability of Rheb to activate mTORC1 and hence it is plausible that PDE4DIP (also called myomegalin) impacts on this relationship, as an increased interaction between Rheb and mTOR should promote growth. On chromosome 13, another growth and differentiation gene, Lmo7, was identified. Lmo7 impacts on myoblast differentiation, being required to induce Pax3 and MyoD expression [79]. Lmo7 is positively associated with age in muscle, while we have previously identified PAX3 as an up-stream transcriptional regulator of the EET induced transcriptome [29], highlighting further why the age-related transcriptome is not simply the opposite of exercise training. i.e., muscle ageing is not simply inactivity and thus is unlikely to be reversed by only activity (this does not at all contest that muscle function can be substantially retained by physical training in many but not all subjects).

### Perspectives and limitations

Over the past 2 decades, therapeutic advances for complex chronic diseases have failed to generate all the progress predicted by the emergence of genetic technologies [80]. Part of the reason for this is that the investment in forward genetic pre-clinical models [81] has not yielded the expected validation of drug targets and it is now widely accepted that the clinical approach to chronic-disease management will have to reflect on numerous interactions between environmental and molecular factors [82]. Thus, an alternative approach, whereby identification of disease networks directly observed in clinical populations may have merit and lead to a more rapid translation of basic science [3], [4], [34], [40], [83]. Validation of such observations is dependent on access to sets of *independent* clinical data sufficiently large to have robust statistical power and diverse enough to be able to generalize the conclusions. It is safe to say that our approach is not at all universally favoured, as there is still a great reliance on so-called ‘validation’ studies involving forward and reverse genetic strategies in mice and cells. We suspect that such validation studies will, in the end prove to be context dependent and no easier to interpret than our *in vivo* molecular studies.

The singular advantage of our approach is that our data is generated under the precise conditions that would ultimately require therapeutic intervention. However, in the present analysis we focus on components of dynamic function, namely muscle mass, as the study of muscle performance will require determination of a wider range of muscle functional parameters (power, torque, velocity) and larger clinical studies, studies which do not exist at present. We also appreciate that our current observations would benefit greatly from follow-on genetic association studies in humans and by pharmacological or nutritional intervention studies. For example we have shown that a simple relationship between more mTOR activation and muscle growth does not exist *in vivo* in humans, albeit we are relying in part on the pharmacological activity of rapamycin to support this observation (off target effects may be present) and do not yet have kinetic data to understand the dynamic nature of this new relationship. We also failed to find a link between inter-subject variation in acute phospho-protein anabolic signalling and gains in lean mass. This may reflect the choice of time-point that we profiled the protein responses under. However, it must also be recognised that quantification of protein abundance changes suffers from numerous complications, including compression or exaggeration of dynamic range and challenges with specificity. Time will tell if our systems-biology translational medicine approach [84] exceeds the performance of traditional approaches taken to yield new therapeutic advances for human health.

## Materials and Methods

This manuscript relies on several microarray studies to produce and independently validate molecular associations. We present a new 89-chip analysis (44 paired samples and 1 baseline-only sample) combined with direct comparisons to literature-based microarray data [23], [24]. The Trappe Lab chip data (GSE28422) was processed using identical methods to our new clinical data. Our QC analysis identified a number of chips that should be removed prior to statistical analysis. Inclusion of such data can both increase or decrease detection of differential gene expression (derived from technical and not biological variation). The analysis as a whole represents the most comprehensive examination of human molecular exercise-ageing pathways, and the application of independent chip resources provides a gold-standard level of validation.

### Derby RET (DRET) subject characteristics

Subjects were recruited from an age range of 18 to 75 y. Before beginning the study all subjects were screened using a medical questionnaire, physical examination and resting ECG with exclusions for overt muscle wasting (>2 SD below age norms) [85], metabolic, respiratory/cardiovascular disorders or other major contraindications to a healthy status. All subjects had normal blood chemistry and were normotensive (BP<140/90). All subjects performed routine activities of daily living and recreation but did not participate in moderate to high intensity aerobic exercise and none had participated in RET in the last 24 months. Body composition was measured at screening and following RET by dual energy X-ray absorptiometry (DEXA) (Lunar Prodigy II, GE Medical Systems). Subject positioning on the DEXA bed was optimized to allow the region of interest (ROI) body compartments to be analyzed separately. The upper leg ROI was selected as the area inferior to the lowest visible point of the coccyx to the mid-point of the patella. All subjects gave their written, informed consent to participate after all procedures and risks were explained. This study was approved by the University of Nottingham Medical School Ethics Committee and complied with the Declaration of Helsinki. The clinical data from these subjects were first reported in 2012 [56]. For the purposes of this article a total of 45 from the original 51 subjects were utilised as this represents the total number of gene-chip profiles that passed the appropriate quality control processes (N = 89 U133+2 Affymetrix chips).

### DRET protocol

The 20-wk fully supervised RET programme was designed to achieve skeletal muscle hypertrophy. Subjects trained three times per week, with each session lasting approximately 60 min. During four weeks of induction training (to ensure adoption and adherence to correct technique) intensity was increased from 40% to 60% 1-RM. For the remaining 16-wk of training intensity was set at 70% 1-RM with multiple sets of 12 repetitions, with two min rest between sets. 1-repetition maximum (1-RM) assessments were made every four weeks to ensure that the intensity of loading was constant. Subjects were excluded from the study for non-compliance, defined as: non-attendance for >6 consecutive sessions, less than 75% attendance, or failure to complete the set exercise regime on >15% attendance. Muscle biopsies (∼150 mg) were taken under fasted-non-exercised (“basal”) and optimal growth conditions (acute exercise-fed conditions, 2.5 h after a single bout of exercise) both before and after chronic-RET from the *vastus lateralis* muscle under local anaesthesia (2% lidocaine, with the use of a conchotome biopsy forceps, as previously described [56]). Blood was collected in pre-chilled tubes containing Lithium Heparin, plasma was separated by centrifugation and was then stored at −80°C until analyses. Plasma glucose concentration was measured on a clinical chemistry glucose analyser (ILAB 300 Plus). For insulin, blood was collected in pre-chilled tubes containing EDTA, plasma was separated by centrifugation within 30 min of collection and was then stored at −80°C until final analyses. Plasma insulin concentration was determined using high sensitivity insulin ELISA systems (Immunodiagnostic systems limited).

### Immuno-blotting

Muscle biopsies (∼20 mg) were homogenized in ice-cold extraction buffer (10 µL.mg^−1^) containing 50 mM Tris-HCl (pH 7.4), 0.1% Triton X-100, 1 mM EDTA, 1 mM EGTA, 50 mM NaF, 0.5 mM activated sodium orthovanadate (Sigma Aldrich, Poole, UK) and a complete protease inhibitor cocktail tablet (Roche, West Sussex, UK). Homogenates were centrifuged at 10,000 *g* for 10 min at 4°C. Bradford assays were used to determine supernatant protein concentrations after which samples were standardized to 1 mg.mL^−1^ in Laemmli loading buffer. Samples were heated at 95°C for 5 min before 15 µg of protein/lane was loaded on to Criterion XT Bis-Tris 12% SDS-PAGE gels (Bio-Rad, Hemel Hempstead, UK) for electrophoresis at 200 V for ∼60 min. Gels were equilibrated in transfer buffer (25 mM Tris, 192 mM glycine, 10% methanol) for 30 min before proteins were electro-blotted on to 0.2 µm PVDF membranes (Bio-Rad) at 100 V for 30 min. After blocking with 5% low-fat milk in TBS-T (Tris-Buffered Saline and 0.1% Tween-20; both Sigma-Aldrich, Poole, UK) for 1 h, membranes were rotated overnight with primary antibody (all AbCam, Cambridge, UK) against our target proteins (AKT, mTOR, p70S6K1, 4EBP1, eEF2) at a concentration of 1∶2000 at 4°C. Membranes were washed (3×5 min) with TBS-T and incubated for 1 h at room temperature with HRP-conjugated anti-rabbit secondary antibody (New England Biolabs, UK), before further washing (3×5 min) with TBS-T and incubation for 5 min with ECL (Immunstar; Bio-Rad). Blots were imaged and quantified by assessing peak density, after ensuring bands were within the linear range of detection using the Chemidoc XRS system (Bio-Rad, Hemel Hempstead, UK). Protein phosphorylation was corrected for loading to actin loading control before the protein signals were subject to PCA to explore relationships between ‘anabolic signals’, specifically in terms of muscle hypertrophy responsiveness.

### RNA extraction and chip processing

Total RNA was isolated from muscle biopsies taken before and after (72 h after the final training session) RET by chloroform-phenol based extraction. In brief, paired tissue samples (obtained before and after RET) of ∼20 mg each were processed simultaneously in 1 mL TRIzol (Invitrogen) using a Mini-Beadbeater-8 (Biospec Inc.) for 15 sec on the “homogenize” setting. After 5 min of incubation at room temperature, 200 µL of chloroform (Sigma-Aldrich) was added and samples shaken vigorously by hand. Samples were briefly incubated on ice prior to centrifugation at 12,000 *g* for 15 min. The supernatant was removed and mixed with isopropanol (Sigma-Aldrich) and spun once more at 12,000 *g* for 10 min, after 10 min of incubation. After a single washing step with 75% EtOH RNA pellets were re-suspended in 40 µL DEPC-treated water (Ambion) and quantified using a NanoDrop Spectrophotometer (NanoDrop Technologies). RNA purity was assessed using the ***A***_260_/***A***_280_, ***A***_260_/***A***_230_ ratios and stored at −80°C. Samples were put through this process in pairs (pre-post samples) while the order of subject processing was carried out to distribute ‘non-responders’ equally.

Reverse transcription of RNA was carried out using the Affymetrix 3′ IVT express kit. 100 ng of total RNA was reverse transcribed as per manufacturer's protocol, and quantified using a Nanodrop ND-1000 instrument. aRNA was fragmented and labeled as per manufacturers protocol and hybridized to Affymetrix U133+2 arrays (Affymetrix, USA). Arrays were washed, stained and scanned following Affymetrix standard procedures, using an Affymetrix 3000 7G scanner and Affymetrix 450 wash station. A visual inspection of each array was carried out.

### Microarray analysis and bioinformatic procedures

Low-level processing of all arrays was undertaken using Bioconductor in R. The Affy package was used to carry out MAS5 based normalization and generate present, marginal and absent (PA) scores. NUSE plots were generated and combined with PCA, outlier samples were identified where both the NUSE plot and PCA was supportive of its exclusion (∼2% of arrays). For baseline correlation analysis, all samples that passed QC were utilized (N = 45). This procedure was applied to the data-set originating from the Trappe laboratory (GSE28422, [24]) and outliers removed from the dataset that failed the QC process, leaving n = 96 for analysis. Pre-exercise training muscle biopsy samples from the HERITAGE family study (N = 50) were also analyzed to yield a second independent data set with a continuous span of age-ranges (see below). The Trappe and HERITAGE datasets therefore represent independent datasets which we utilized, where possible, to validate the pathway analysis of our study. Such confirmation benchmarks results using thousands of data-points and is more desirable that targeted real-time qPCR confirmation (where the gene selection is biased and the sample size inappropriate to make statistical conclusions). Annotation of all CEL files used ‘hgu133plus2cdf_2.9.1.tgz’ while annotation of probe-set lists was then updated using the Ingenuity Pathway Analysis database, as of August 2012.

Our first objective was to identify the gene-networks regulated in *proportion* to gains in upper leg muscle mass (hypertrophy), the same location as our biopsy sample. Such analysis relies on the established principal that adaptation responses (for the majority of phenotypes) to exercise training in outbred populations is highly variable, typically reflecting genetic and epigenetic variation and in genomic variation. We utilized quantitative SAM analysis [3], [36] to generate a list of genes which vary in a positive and negative manner with changes in DEXA assessed upper leg lean mass. This was applied to PA filtered data and the statistical parameter generated is a q-value (false discovery rate). This provided for the first time a candidate list of gene-changes that may exhibit primary or secondary influence over muscle growth in humans. The gene-list was then subject to IPA based pathway analysis and in particular the *Upstream Analysis* tool in IPA was utilized. This analysis has similarities to the Molecular Connectivity Database [86] where pre-existing collections of RNA signatures are compared with our lean-mass related gene list, and significant overlaps identified. An overlap *P*-value is generated based on the degree of overlap between the gene-set within the IPA database (which reflects the RNA molecules changed in response to a ‘mediator’ such as a transcription factor or a drug) and our data set, adjusting for data set sizes using the Fischer's Exact Test. We accepted a stringent *P*-value of p<0.001 as being significant. A second parameter is the activation “z-score” where the directional change in RNA is compared between the IPA mediator data-set and our lean-mass gain gene list. The z-score informs on whether the drug/protein mediator is likely to be ‘active’ or ‘inhibited’ during gains in lean mass. Thus, if we discovered that an *antagonist* is ‘inhibited’ in our analysis, this indicates that the drug target is activated. However, in the present study the data-input refers to genes, which positively or negatively correlate with lean mass gains e.g. if we find a “Statin” signature was inhibited, it is interpreted that HMG-CoA reductase regulated genes are negatively correlated with lean mass gain.

The two-step process presented above generates a focused gene-list with a high statistical rigor for true positive associations. This type of analysis also utilizes the full range of physiological response observed, however it assumes that expression of important genes will relate in a linear manner to lean mass gain and thus can not discover all appropriate associations. We then contextualize the statistical findings both in terms of subject characteristics and through comparison of the response of these significant networks with independent gene-array data (e.g. [24]). At this stage we utilized descriptive statistics, plotting the significant network genes as simple expression values relative to the quartile distribution of lean-mass gains to allow for clear discussion of the results. As these plots are based on the z-scores and *P*-values as above, no further statistical analysis is presented.

Following identification of our primary objectives we then carried out a classic differential expression analysis using SAM. Given that we have established that *chronic* differential expression patterns, following exercise training, are dependent on the presence of physiological adaptation we removed 6 subjects that demonstrated no gain in lean mass. This yielded a list of differentially expressed genes that could then be compared with the RET gene-list generated from the Trappe laboratory data and our published exercise studies [24]. Secondary analysis, where subject age or baseline lean-mass was related to baseline gene-expression was carried out using quantitative SAM analysis as described above [3], [36]. This allowed us to present comparisons of the RET gene-list with other modes of exercise, such as endurance exercise training or disease [3], [29] and age-related analysis [23], [24]. PCA was utilized to visualize the association between selected physiological and protein expression parameters and training induced changes in muscle lean mass. PCA was implemented in R, using prcomp() command, which calculated a singular value decomposition and plots the selected principal components using the plot command in R. All data was individually transformed to a median value within that data set so that all variables were within a consistent data range. In each case the majority (∼65%) of the total variance was captured by the first two principal components.

Finally, positional gene enrichment analysis (PGE) was used to identify whether the classification genes (or the classifier network genes) were significantly enriched within given chromosomal regions [43]. This analysis is based on the following rules: Rule 1: it contains at least two genes of interest, Rule 2: there is no smaller region containing the same genes of interest, Rule 3: there is no bigger region with more genes of interest and the same genes not of interest, Rule 4: there is no larger encompassing region with a higher percentage of genes of interest, Rule 5: there is no smaller encompassed region with a better *P*-value, Rule 6: it does not contain any region having less than expected genes of interest. The approach of PGE exhaustively evaluates the over-representation at all chromosomal resolution levels simultaneously.

## Supporting Information

Dataset S1 Exercise regulated genes from multiple studies.
(XLSX)

Dataset S2 Genes regulated in proportion to lean mass gains in DRET.
(XLSX)

Dataset S3 Genes that co-vary with human age across DRET and HERITAGE.
(XLSX)

Dataset S4 Results from the connectivity map analysis for drugs that regulate age-related genes.
(XLSX)

Dataset S5 Chromosomal localization analysis.
(XLSX)

Figure S1 Gene networks activated through inhibition of a repressor molecule AHR or KRAS following 20 wk RET A) KRAS Z = −2.8 p = 3.88E-04 and AHR Z = −2.4 p = 2.96E-07 B) 6 wk of EET KRAS Z = −2.5 p = 1.92E-11 and Z = −2.9 p = 9.66E-14.
(TIF)

Figure S2 Canonical pathways associated with lean muscle mass gains derived from IPA database.
(TIF)

Figure S3 Canonical pathways associated with age derived from IPA database.
(TIF)

Figure S4 Gene networks positively associated with age in human muscle contain RNA's which are not regulated by either A) acute resistance ET (Trappe data-set GSE28422) nor B) 6 wk endurance exercise training (Keller et al 2011 JAP).
(TIF)

Figure S5 Age related gene networks where the central node is inhibited with age contain RNA's which are positively correlated with age and increased with A) acute resistance ET (Trappe data-set GSE28422) and B) 6 wk endurance exercise training (Keller et al 2011 JAP) providing a clear indication that age and exercise are not simple ‘opposites’ at the molecular level.
(TIF)

## References

[1] Atherton PJ, Smith K (2012) Muscle protein synthesis in response to nutrition and exercise. The Journal of physiology 590: 1049–1057. 22289911 10.1113/jphysiol.2011.225003 PMC3381813

[2] Bouchard C, Rankinen T, Timmons JA (2011) Genomics and Genetics in the Biology of Adaptation to Exercise. Comprehensive Physiology 1603–1648. 23733655 10.1002/cphy.c100059 PMC3938186

[3] Stephens NA, Gallagher IJ, Rooyackers O, Skipworth RJ, Tan BH, et al (2010) Using transcriptomics to identify and validate novel biomarkers of human skeletal muscle cancer cachexia. Genome Med 2: 1. 20193046 10.1186/gm122 PMC2829926

[4] Gallagher IJ, Stephens NA, MacDonald AJ, Skipworth RJE, Husi H, et al (2012) Suppression of skeletal muscle turnover in cancer cachexia: evidence from the transcriptome in sequential human muscle biopsies. Clinical cancer research: an official journal of the American Association for Cancer Research 18: 2817–2827. 22452944 10.1158/1078-0432.CCR-11-2133

[5] Glover EI, Phillips SM, Oates BR, Tang JE, Tarnopolsky MA, et al (2008) Immobilization induces anabolic resistance in human myofibrillar protein synthesis with low and high dose amino acid infusion. The Journal of physiology 586: 6049–6061. 18955382 10.1113/jphysiol.2008.160333 PMC2655417

[6] Gustafsson T, Osterlund T, Flanagan JN, Von Waldén F, Trappe TA, et al (2010) Effects of 3 days unloading on molecular regulators of muscle size in humans. Journal of applied physiology (Bethesda, Md: 1985) 109: 721–727. 10.1152/japplphysiol.00110.2009 20538844

[7] Kumar V, Selby A, Rankin D, Patel R, Atherton P, et al (2009) Age-related differences in the dose-response relationship of muscle protein synthesis to resistance exercise in young and old men. The Journal of physiology 587: 211–217. 19001042 10.1113/jphysiol.2008.164483 PMC2670034

[8] Mitchell CJ, Churchward-Venne TA, West DWD, Burd NA, Breen L, et al (2012) Resistance exercise load does not determine training-mediated hypertrophic gains in young men. Journal of applied physiology (Bethesda, Md: 1985) 113: 71–77. 10.1152/japplphysiol.00307.2012 PMC3404827 22518835

[9] Churchward-Venne TA, Burd NA, Phillips SM (2012) Research Group EM (2012) Nutritional regulation of muscle protein synthesis with resistance exercise: strategies to enhance anabolism. Nutrition & metabolism 9: 40. 22594765 10.1186/1743-7075-9-40 PMC3464665

[10] Rommel C, Bodine SC, Clarke BA, Rossman R, Nunez L, et al (2001) Mediation of IGF-1-induced skeletal myotube hypertrophy by PI(3)K/Akt/mTOR and PI(3)K/Akt/GSK3 pathways. Nature cell biology 3: 1009–1013. 11715022 10.1038/ncb1101-1009

[11] Iadevaia V, Huo Y, Zhang Z, Foster LJ, Proud CG (2012) Roles of the mammalian target of rapamycin, mTOR, in controlling ribosome biogenesis and protein synthesis. Biochemical Society transactions 40: 168–172. 22260684 10.1042/BST20110682

[12] Terzis G, Georgiadis G, Stratakos G, Vogiatzis I, Kavouras S, et al (2008) Resistance exercise-induced increase in muscle mass correlates with p70S6 kinase phosphorylation in human subjects. Eur J Appl Physiol 102: 145–152. 17874120 10.1007/s00421-007-0564-y

[13] Drummond MJ, Miyazaki M, Dreyer HC, Pennings B, Dhanani S, et al (2009) Expression of growth-related genes in young and older human skeletal muscle following an acute stimulation of protein synthesis. Journal of applied physiology (Bethesda, Md: 1985) 106: 1403–1411. 10.1152/japplphysiol.90842.2008 PMC2698637 18787087

[14] Dickinson JM, Fry CS, Drummond MJ, Gundermann DM, Walker DK, et al (n.d.) Mammalian target of rapamycin complex 1 activation is required for the stimulation of human skeletal muscle protein synthesis by essential amino acids. J Nutr 141: 856–862. 21430254 10.3945/jn.111.139485 PMC3077888

[15] Fry CS, Drummond MJ, Glynn EL, Dickinson JM, Gundermann DM, et al (2011) Aging impairs contraction-induced human skeletal muscle mTORC1 signaling and protein synthesis. Skeletal muscle 1: 11. 21798089 10.1186/2044-5040-1-11 PMC3156634

[16] Li M, Verdijk LB, Sakamoto K, Ely B, Van Loon LJC, et al (2012) Reduced AMPK-ACC and mTOR signaling in muscle from older men, and effect of resistance exercise. Mechanisms of ageing and development 133: 655–664. 23000302 10.1016/j.mad.2012.09.001 PMC3631591

[17] Farnfield MM, Breen L, Carey KA, Garnham A, Cameron-Smith D (2012) Activation of mTOR signalling in young and old human skeletal muscle in response to combined resistance exercise and whey protein ingestion. Applied physiology, nutrition, and metabolism = Physiologie appliquée, nutrition et métabolisme 37: 21–30. 10.1139/h11-132 22148961

[18] West DWD, Kujbida GW, Moore DR, Atherton P, Burd NA, et al (2009) Resistance exercise-induced increases in putative anabolic hormones do not enhance muscle protein synthesis or intracellular signalling in young men. J Physiol 587: 5239–5247. 19736298 10.1113/jphysiol.2009.177220 PMC2790261

[19] Spangenburg EE, Le Roith D, Ward CW, Bodine SC (2008) A functional insulin-like growth factor receptor is not necessary for load-induced skeletal muscle hypertrophy. The Journal of physiology 586: 283–291. 17974583 10.1113/jphysiol.2007.141507 PMC2375552

[20] Goodman CA, Frey JW, Mabrey DM, Jacobs BL, Lincoln HC, et al (2011) The role of skeletal muscle mTOR in the regulation of mechanical load-induced growth. The Journal of physiology 589: 5485–5501. 21946849 10.1113/jphysiol.2011.218255 PMC3240886

[21] Camera DM, Edge J, Short MJ, Hawley JA, Coffey VG (2010) Early time course of Akt phosphorylation after endurance and resistance exercise. Medicine and science in sports and exercise 42: 1843–1852. 20195183 10.1249/MSS.0b013e3181d964e4

[22] Timmons JA (2011) Variability in training-induced skeletal muscle adaptation. J Appl Physiol 110: 846–853. 21030666 10.1152/japplphysiol.00934.2010 PMC3069632

[23] Melov S, Tarnopolsky MA, Beckman K, Felkey K, Hubbard A (2007) Resistance exercise reverses aging in human skeletal muscle. PLoS ONE 2: e465 doi:10.1371/journal.pone.0000465. 17520024 10.1371/journal.pone.0000465 PMC1866181

[24] Raue U, Trappe TA, Estrem ST, Qian H-R, Helvering LM, et al (2012) Transcriptome signature of resistance exercise adaptations: mixed muscle and fiber type specific profiles in young and old adults. Journal of applied physiology (Bethesda, Md: 1985) 112: 1625–1636. 10.1152/japplphysiol.00435.2011 PMC3365403 22302958

[25] Bell R, Hubbard A, Chettier R, Chen D, Miller JP, et al (2009) A human protein interaction network shows conservation of aging processes between human and invertebrate species. PLoS Genet 5: e1000414 doi:10.1371/journal.pgen.1000414. 19293945 10.1371/journal.pgen.1000414 PMC2657003

[26] Welle S, Brooks AI, Delehanty JM, Needler N, Bhatt K, et al (2004) Skeletal muscle gene expression profiles in 20–29 year old and 65–71 year old women. Exp Gerontol 39: 369–377. 15036396 10.1016/j.exger.2003.11.011

[27] Giresi PG, Stevenson EJ, Theilhaber J, Koncarevic A, Parkington J, et al (2005) Identification of a molecular signature of sarcopenia. Physiol Genomics 21: 253–263. 15687482 10.1152/physiolgenomics.00249.2004

[28] Davidsen PK, Gallagher IJ, Hartman JW, Tarnopolsky MA, Dela F, et al (2011) High responders to resistance exercise training demonstrate differential regulation of skeletal muscle microRNA expression. J Appl Physiol 110: 309–317. 21030674 10.1152/japplphysiol.00901.2010

[29] Keller P, Vollaard NB, Gustafsson T, Gallagher IJ, Sundberg CJ, et al (2011) A transcriptional map of the impact of endurance exercise training on skeletal muscle phenotype. J Appl Physiol 110: 46–59. 20930125 10.1152/japplphysiol.00634.2010 PMC3253010

[30] Boule NG, Weisnagel SJ, Lakka TA, Tremblay A, Bergman RN, et al (2005) Effects of exercise training on glucose homeostasis: the HERITAGE Family Study. Diabetes Care 28: 108–114. 15616242 10.2337/diacare.28.1.108

[31] Timmons Ja, Jansson E, Fischer H, Gustafsson T, Greenhaff PL, et al (2005) Modulation of extracellular matrix genes reflects the magnitude of physiological adaptation to aerobic exercise training in humans. BMC biology 3: 19. A. 16138928 10.1186/1741-7007-3-19 PMC1224855

[32] Vollaard NB, Constantin-Teodosiu D, Fredriksson K, Rooyackers O, Jansson E, et al (2009) Systematic analysis of adaptations in aerobic capacity and submaximal energy metabolism provides a unique insight into determinants of human aerobic performance. J Appl Physiol 106: 1479–1486. 19196912 10.1152/japplphysiol.91453.2008

[33] Keller P, Vollaard N, Babraj J, Ball D, Sewell DA, et al (2007) Using systems biology to define the essential biological networks responsible for adaptation to endurance exercise training. Biochem Soc Trans 35: 1306–1309. 17956337 10.1042/BST0351306

[34] Timmons JA, Knudsen S, Rankinen T, Koch LG, Sarzynski M, et al (2010) Using molecular classification to predict gains in maximal aerobic capacity following endurance exercise training in humans. Journal of applied physiology 108: 1487–1496. 20133430 10.1152/japplphysiol.01295.2009 PMC2886694

[35] FELCIANO RM, BAVARI S, RICHARDS DR, BILLAUD J-N, WARREN T, et al (2013) Predictive systems biology approach to broad-spectrum, host-directed drug target discovery in infectious diseases. Pacific Symposium on Biocomputing 18: 17–28 Available: http://psb.stanford.edu/psb-online/proceedings/psb13/felciano.pdf 23424108

[36] Tusher VG, Tibshirani R, Chu G (2001) Significance analysis of microarrays applied to the ionizing radiation response. Proc Natl Acad Sci U S A 98: 5116–5121. 11309499 10.1073/pnas.091062498 PMC33173

[37] Atherton PJ, Babraj J, Smith K, Singh J, Rennie MJ, et al (2005) Selective activation of AMPK-PGC-1alpha or PKB-TSC2-mTOR signaling can explain specific adaptive responses to endurance or resistance training-like electrical muscle stimulation. Faseb J 19: 786–788. 15716393 10.1096/fj.04-2179fje

[38] Gustafson WC, Weiss WA (2010) Myc proteins as therapeutic targets. Oncogene 29: 1249–1259. 20101214 10.1038/onc.2009.512 PMC2904682

[39] Welle S, Brooks AI, Delehanty JM, Needler N, Thornton CA (2003) Gene expression profile of aging in human muscle. Physiol Genomics 14: 149–159. 12783983 10.1152/physiolgenomics.00049.2003

[40] Gallagher I (2010) Integration of microRNA changes in vivo identifies novel molecular features of muscle insulin resistance in Type 2 Diabetes. Genome Med 2: 9. 20353613 10.1186/gm130 PMC2847700

[41] Timmons JA, Norrbom J, Scheele C, Thonberg H, Wahlestedt C, et al (2006) Expression profiling following local muscle inactivity in humans provides new perspective on diabetes-related genes. Genomics 87: 165–172. 16326070 10.1016/j.ygeno.2005.09.007

[42] Bouchard C, Leon AS, Rao DC, Skinner JS, Wilmore JH, et al (1995) The HERITAGE family study. Aims, design, and measurement protocol. Med Sci Sports Exerc 27: 721–729. 7674877

[43] De Preter K, Barriot R, Speleman F, Vandesompele J, Moreau Y (2008) Positional gene enrichment analysis of gene sets for high-resolution identification of overrepresented chromosomal regions. Nucleic acids research 36: e43. 18346969 10.1093/nar/gkn114 PMC2367735

[44] Timmons JA, Baar K, Davidsen PK, Atherton PJ (2012) Is irisin a human exercise gene? Nature 488: E9–E10. 22932392 10.1038/nature11364

[45] Kadi F, Ponsot E (2010) The biology of satellite cells and telomeres in human skeletal muscle: effects of aging and physical activity. Scandinavian journal of medicine & science in sports 20: 39–48. 10.1111/j.1600-0838.2009.00966.x 19765243

[46] McCall GE, Byrnes WC, Dickinson A, Pattany PM, Fleck SJ (1996) Muscle fiber hypertrophy, hyperplasia, and capillary density in college men after resistance training. Journal of applied physiology (Bethesda, Md: 1985) 81: 2004–2012. 10.1152/jappl.1996.81.5.2004 8941522

[47] Holm L, Van Hall G, Rose AJ, Miller BF, Doessing S, et al (2010) Contraction intensity and feeding affect collagen and myofibrillar protein synthesis rates differently in human skeletal muscle. American journal of physiology Endocrinology and metabolism 298: E257–69. 19903866 10.1152/ajpendo.00609.2009

[48] Halevy O, Lerman O (1993) Retinoic acid induces adult muscle cell differentiation mediated by the retinoic acid receptor-alpha. Journal of cellular physiology 154: 566–572. 8382210 10.1002/jcp.1041540315

[49] Bunaciu RP, Yen A (2011) Activation of the aryl hydrocarbon receptor AhR Promotes retinoic acid-induced differentiation of myeloblastic leukemia cells by restricting expression of the stem cell transcription factor Oct4. Cancer research 71: 2371–2380. 21262915 10.1158/0008-5472.CAN-10-2299 PMC3391168

[50] McKay BR, O'Reilly CE, Phillips SM, Tarnopolsky MA, Parise G (2008) Co-expression of IGF-1 family members with myogenic regulatory factors following acute damaging muscle-lengthening contractions in humans. The Journal of physiology 586: 5549–5560. 18818249 10.1113/jphysiol.2008.160176 PMC2655389

[51] Parise G, McKinnell IW, Rudnicki MA (2008) Muscle satellite cell and atypical myogenic progenitor response following exercise. Muscle & nerve 37: 611–619 Available: http://www.ncbi.nlm.nih.gov/pubmed/18351585 Accessed 12 October 2012. 18351585 10.1002/mus.20995

[52] Ge Y, Sun Y, Chen J (2011) IGF-II is regulated by microRNA-125b in skeletal myogenesis. The Journal of cell biology 192: 69–81. 21200031 10.1083/jcb.201007165 PMC3019547

[53] Saito A, Sugawara A, Uruno A, Kudo M, Kagechika H, et al (2007) All-trans retinoic acid induces in vitro angiogenesis via retinoic acid receptor: possible involvement of paracrine effects of endogenous vascular endothelial growth factor signaling. Endocrinology 148: 1412–1423. 17170094 10.1210/en.2006-0900

[54] Johnston APW, Baker J, Bellamy LM, McKay BR, De Lisio M, et al (2010) Regulation of muscle satellite cell activation and chemotaxis by angiotensin II. PLoS ONE 5: e15212 doi:10.1371/journal.pone.0015212. 21203566 10.1371/journal.pone.0015212 PMC3006204

[55] Gorski DH, Walsh K (2000) The Role of Homeobox Genes in Vascular Remodeling and Angiogenesis. Circulation Research 87: 865–872. 11073881 10.1161/01.res.87.10.865

[56] Phillips B, Williams J, Atherton PJ, Smith K, Hildebrandt W, et al (2011) Resistance exercise training improves age-related declines in leg vascular conductance and rejuvenates acute leg blood flow responses to feeding and exercise. Journal of applied physiology (Bethesda, Md: 1985) 112: 347–353. 10.1152/japplphysiol.01031.2011 21998269

[57] Gustafsson T, Puntschart A, Kaijser L, Jansson E, Sundberg CJ (1999) Exercise-induced expression of angiogenesis-related transcription and growth factors in human skeletal muscle. Am J Physiol 276: H679–85. 9950871 10.1152/ajpheart.1999.276.2.H679

[58] Axel D (2001) All-trans retinoic acid regulates proliferation, migration, differentiation, and extracellular matrix turnover of human arterial smooth muscle cells. Cardiovascular Research 49: 851–862. 11230985 10.1016/s0008-6363(00)00312-6

[59] Ohtake F, Baba A, Takada I, Okada M, Iwasaki K, et al (2007) Dioxin receptor is a ligand-dependent E3 ubiquitin ligase. Nature 446: 562–566. 17392787 10.1038/nature05683

[60] Abdelrahim M, Smith R, Safe S (2003) Aryl hydrocarbon receptor gene silencing with small inhibitory RNA differentially modulates Ah-responsiveness in MCF-7 and HepG2 cancer cells. Molecular pharmacology 63: 1373–1381. 12761348 10.1124/mol.63.6.1373

[61] Ameln H, Gustafsson T, Sundberg CJ, Okamoto K, Jansson E, et al (2005) Physiological activation of hypoxia inducible factor-1 in human skeletal muscle. Faseb J 19: 1009–1011. 15811877 10.1096/fj.04-2304fje

[62] Andreasen EA, Mathew LK, Löhr CV, Hasson R, Tanguay RL (2007) Aryl hydrocarbon receptor activation impairs extracellular matrix remodeling during zebra fish fin regeneration. Toxicological sciences: an official journal of the Society of Toxicology 95: 215–226. 17003102 10.1093/toxsci/kfl119

[63] Murphy KA, Quadro L, White LA (2007) The intersection between the aryl hydrocarbon receptor (AhR)- and retinoic acid-signaling pathways. Vitamins and hormones 75: 33–67. 17368311 10.1016/S0083-6729(06)75002-6

[64] Bamman MM, Petrella JK, Kim JS, Mayhew DL, Cross JM (2007) Cluster analysis tests the importance of myogenic gene expression during myofiber hypertrophy in humans. J Appl Physiol 102: 2232–2239. 17395765 10.1152/japplphysiol.00024.2007

[65] Moore DR, Del Bel NC, Nizi KI, Hartman JW, Tang JE, et al (2007) Resistance training reduces fasted- and fed-state leucine turnover and increases dietary nitrogen retention in previously untrained young men. J Nutr 137: 985–991. 17374665 10.1093/jn/137.4.985

[66] Von Walden F, Casagrande V, Östlund Farrants A-K, Nader GA (2012) Mechanical loading induces the expression of a Pol I regulon at the onset of skeletal muscle hypertrophy. American journal of physiology Cell physiology 302: C1523–30. 22403788 10.1152/ajpcell.00460.2011

[67] West DWD, Burd NA, Coffey VG, Baker SK, Burke LM, et al (2011) Rapid aminoacidemia enhances myofibrillar protein synthesis and anabolic intramuscular signaling responses after resistance exercise. The American journal of clinical nutrition 94: 795–803 doi:10.3945/ajcn.111.013722. 21795443 10.3945/ajcn.111.013722

[68] Moore DR, Atherton PJ, Rennie MJ, Tarnopolsky MA, Phillips SM (2011) Resistance exercise enhances mTOR and MAPK signalling in human muscle over that seen at rest after bolus protein ingestion. Acta physiologica (Oxford, England) 201: 365–372. 10.1111/j.1748-1716.2010.02187.x 20874802

[69] Atherton PJ, Etheridge T, Watt PW, Wilkinson D, Selby A, et al (2010) Muscle full effect after oral protein: time-dependent concordance and discordance between human muscle protein synthesis and mTORC1 signaling. The American journal of clinical nutrition 92: 1080–1088. 20844073 10.3945/ajcn.2010.29819

[70] Greenhaff PL, Karagounis LG, Peirce N, Simpson EJ, Hazell M, et al (2008) Disassociation between the effects of amino acids and insulin on signaling, ubiquitin ligases, and protein turnover in human muscle. Am J Physiol Endocrinol Metab 295: E595–604. 18577697 10.1152/ajpendo.90411.2008 PMC2536736

[71] Mayhew DL, Kim JS, Cross JM, Ferrando AA, Bamman MM (2009) Translational signaling responses preceding resistance training-mediated myofiber hypertrophy in young and old humans. J Appl Physiol 107: 1655–1662. 19589955 10.1152/japplphysiol.91234.2008 PMC2777794

[72] Kenyon CJ (2010) The genetics of ageing. Nature 464: 504–512. 20336132 10.1038/nature08980

[73] Janssen I, Heymsfield SB, Ross R (2002) Low relative skeletal muscle mass (sarcopenia) in older persons is associated with functional impairment and physical disability. J Am Geriatr Soc 50: 889–896. 12028177 10.1046/j.1532-5415.2002.50216.x

[74] Goodman CA, Miu MH, Frey JW, Mabrey DM, Lincoln HC, et al (2011) A phosphatidylinositol 3-kinase/protein kinase B-independent activation of mammalian target of rapamycin signaling is sufficient to induce skeletal muscle hypertrophy. Mol Biol Cell 21: 3258–3268. 10.1091/mbc.E10-05-0454 PMC2938390 20668162

[75] Baar K, Esser K (1999) Phosphorylation of p70(S6k) correlates with increased skeletal muscle mass following resistance exercise. Am J Physiol 276: C120–7. 9886927 10.1152/ajpcell.1999.276.1.C120

[76] Mahoney DJ, Parise G, Melov S, Safdar A, Tarnopolsky MA (2005) Analysis of global mRNA expression in human skeletal muscle during recovery from endurance exercise. Faseb J 19: 1498–1500. 15985525 10.1096/fj.04-3149fje

[77] Zheng J-Y, Yu D, Foroohar M, Ko E, Chan J, et al (2003) Regulation of the expression of the prostate-specific antigen by claudin-7. The Journal of membrane biology 194: 187–197. 14502431 10.1007/s00232-003-2038-4

[78] Kim HW, Ha SH, Lee MN, Huston E, Kim D-H, et al (2010) Cyclic AMP controls mTOR through regulation of the dynamic interaction between Rheb and phosphodiesterase 4D. Molecular and cellular biology 30: 5406–5420. 20837708 10.1128/MCB.00217-10 PMC2976372

[79] Dedeic Z, Cetera M, Cohen TV, Holaska JM (2011) Emerin inhibits Lmo7 binding to the Pax3 and MyoD promoters and expression of myoblast proliferation genes. Journal of cell science 124: 1691–1702. 21525034 10.1242/jcs.080259

[80] Lipinski C, Hopkins A (2004) Navigating chemical space for biology and medicine. Nature 432: 855–861. 15602551 10.1038/nature03193

[81] Shanks N, Greek R, Greek J (2009) Are animal models predictive for humans? Philosophy, ethics, and humanities in medicine: PEHM 4: 2. 10.1186/1747-5341-4-2 PMC2642860 19146696

[82] Hopkins AL (2008) Network pharmacology: the next paradigm in drug discovery. Nat Chem Biol 4: 682–690. 18936753 10.1038/nchembio.118

[83] Fredriksson K, Tjäder I, Keller P, Petrovic N, Ahlman B, et al (2008) Dysregulation of mitochondrial dynamics and the muscle transcriptome in ICU patients suffering from sepsis induced multiple organ failure. PLoS ONE 3: e3686 doi:10.1371/journal.pone.0003686. 18997871 10.1371/journal.pone.0003686 PMC2579334

[84] Timmons Ja (2011) What happens if you pose the wrong questions? The Journal of physiology 589: 4799–4801. 21965633 10.1113/jphysiol.2011.213413 PMC3213425

[85] Gallagher D, Visser M, De Meersman RE, Sepulveda D, Baumgartner RN, et al (1997) Appendicular skeletal muscle mass: effects of age, gender, and ethnicity. J Appl Physiol 83: 229–239. 9216968 10.1152/jappl.1997.83.1.229

[86] Lamb J, Crawford ED, Peck D, Modell JW, Blat IC, et al (2006) The Connectivity Map: using gene-expression signatures to connect small molecules, genes, and disease. Science 313: 1929–1935. 17008526 10.1126/science.1132939

