# Correction: Molecular Networks of Human Muscle Adaptation to Exercise and Age

**DOI:** 10.1371/annotation/35682594-0e72-496b-8641-f956d22c391e

**Published:** 2013-04-03

**Authors:** Bethan E. Phillips, John P. Williams, Thomas Gustafsson, Claude Bouchard, Tuomo Rankinen, Steen Knudsen, Kenneth Smith, James A. Timmons, Philip J. Atherton

The authors apologise for omitting an important article in the field of human ageing. The data in the article are consistent with their ontology analysis of the human age related genes.

The reference was omitted from the final sentence of the third paragraph under the subheading ‘Age Muscle Gene Networks’ in the Results section. The sentence should read:

“To this end, we generated 52 new U133+2 profiles (17–63 yr) from muscle biopsy samples from the HERITAGE Family Study [42] and were able to identify a set of 580 genes or transcripts (Dataset 3) which were correlated with age in both clinical studies and which was enriched in post-transcriptional (consistent with the work of Harries et al. (2011)) and chronic disease traits (Figure S3) but not mitochondrial related gene-sets.”

The reference is:

Harries LW, Hernandez D, Henley W, Wood AR, Holly AC, et al. (2011) Human aging is characterized by focused changes in gene expression and deregulation of alternative splicing. Aging Cell. 868–78. doi: 10.1111/j.1474-9726.2011.00726.x

In addition, the link to XRgenomics LTD in the Acknowledgments is incorrect. The link should read http://www.XRgenomics.com

