# Correction: Molecular Networks of Human Muscle Adaptation to Exercise and Age

**DOI:** 10.1371/annotation/0dd3671e-1460-48fa-9d6a-2865dce78c07

**Published:** 2013-04-25

**Authors:** Bethan E. Phillips, John P. Williams, Thomas Gustafsson, Claude Bouchard, Tuomo Rankinen, Steen Knudsen, Kenneth Smith, James A. Timmons, Philip J. Atherton

For the avoidance of any misunderstanding on competing interests, the authors declare that Jamie Timmons is a Founding Director and Thomas Gustafsson, Steen Knudsen and Bethan Phillips are share holders at XR Genomics, a Personalised Health and Fitness company. This should have been indicated at the time of publication. No agreements were in place concerning the execution or publication of this work. The other authors have no competing interests to declare.

