# Supplementary figures and images for: Molecular Networks of Human Muscle Adaptation to Exercise and Age
Source: PLoS Genet. 2013 Mar 21;9(3):e1003389. doi: 10.1371/journal.pgen.1003389 (PMC3605101; doi:10.1371/journal.pgen.1003389)

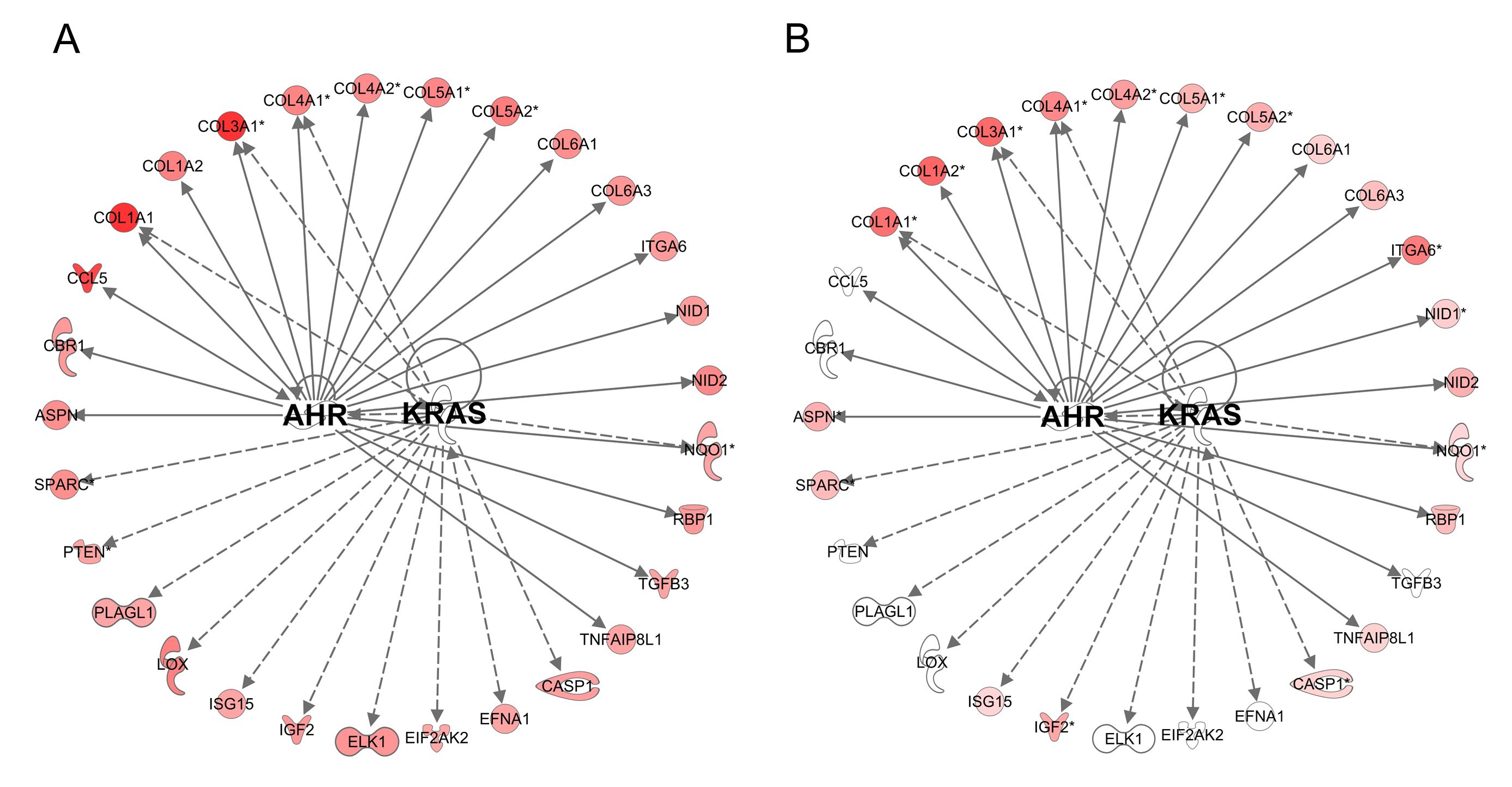

Supplement: Figure S1 — Gene networks activated through inhibition of a repressor molecule AHR or KRAS following 20 wk RET A) KRAS Z = −2.8 p = 3.88E-04 and AHR Z = −2.4 p = 2.96E-07 B) 6 wk of EET KRAS Z = −2.5 p = 1.92E-11 and Z = −2.9 p = 9.66E-14. (TIF) [file pgen.1003389.s006.tif]

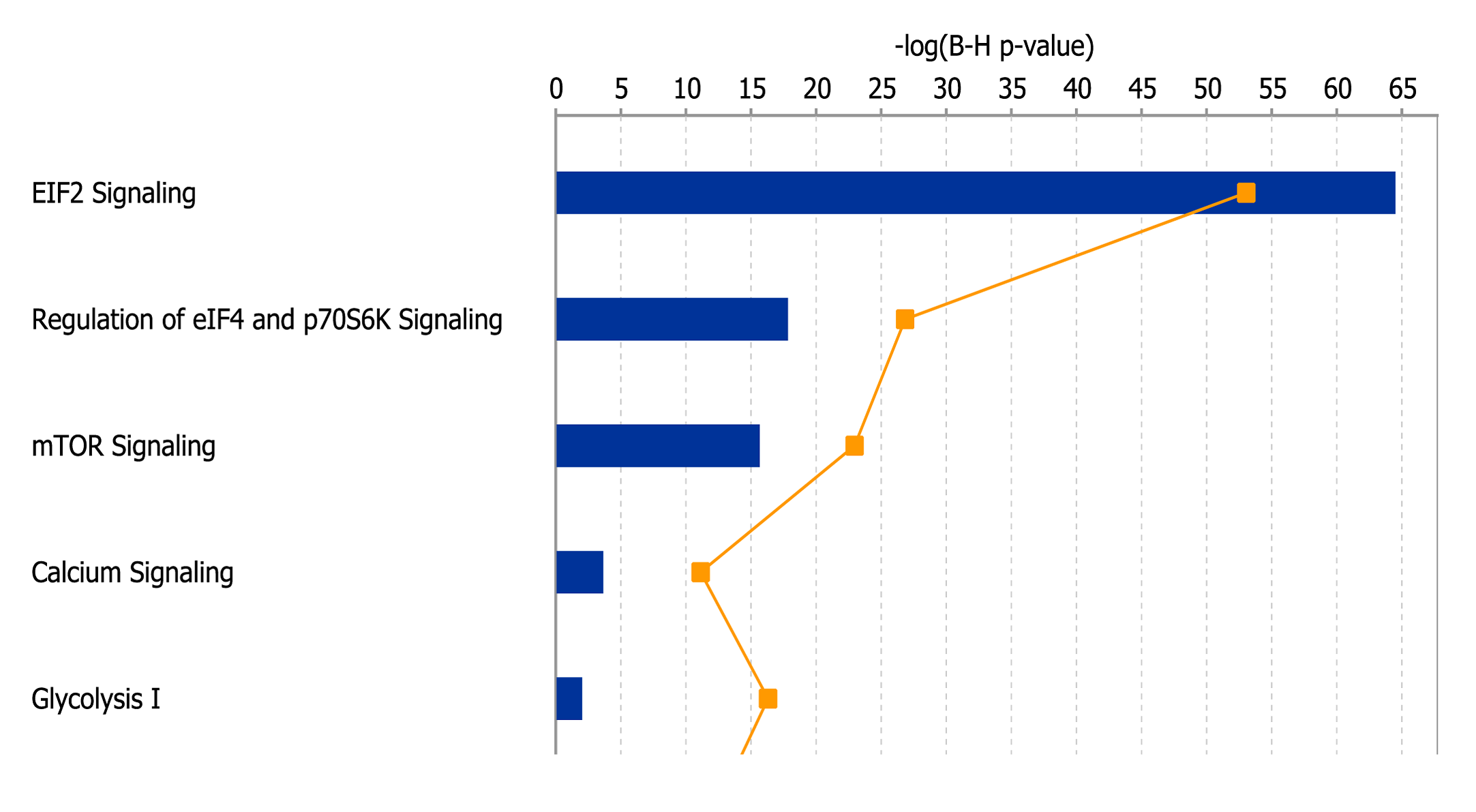

Supplement: Figure S2 — Canonical pathways associated with lean muscle mass gains derived from IPA database. (TIF) [file pgen.1003389.s007.tif]

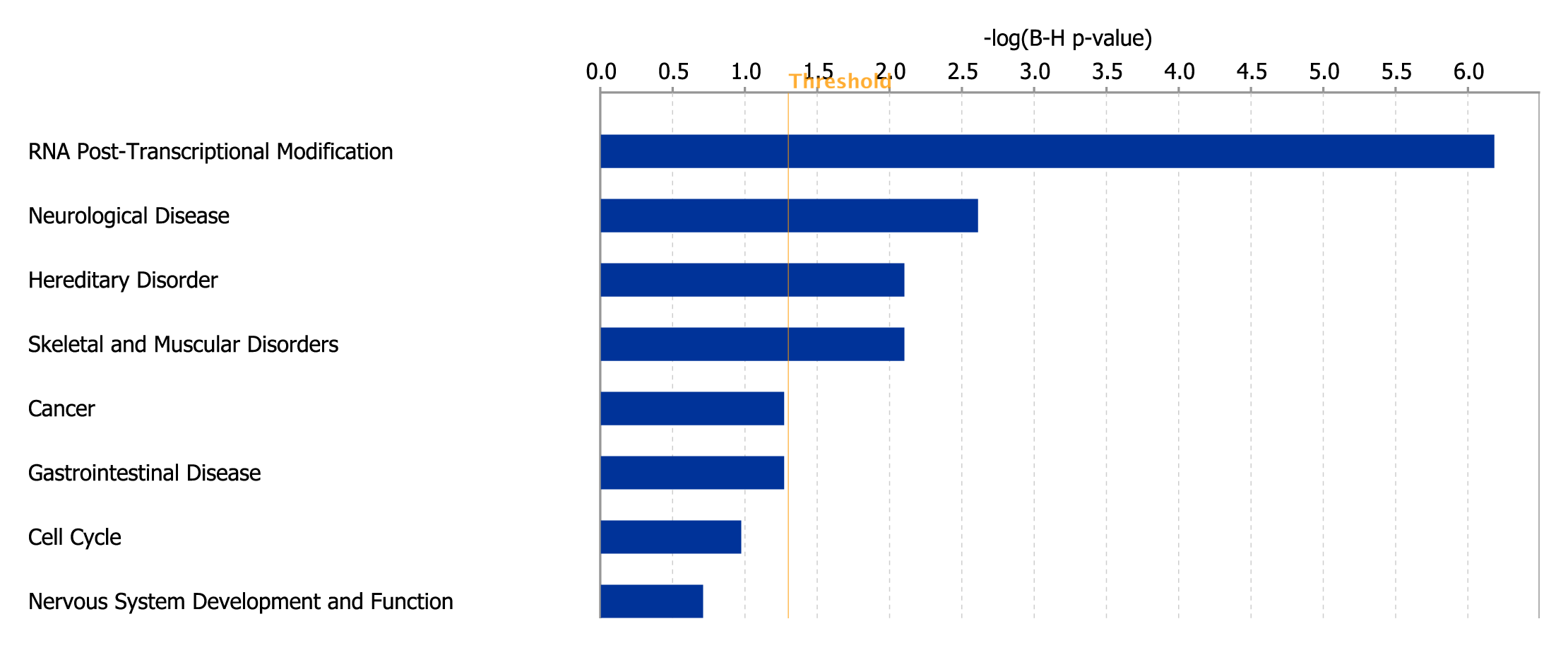

Supplement: Figure S3 — Canonical pathways associated with age derived from IPA database. (TIF) [file pgen.1003389.s008.tif]

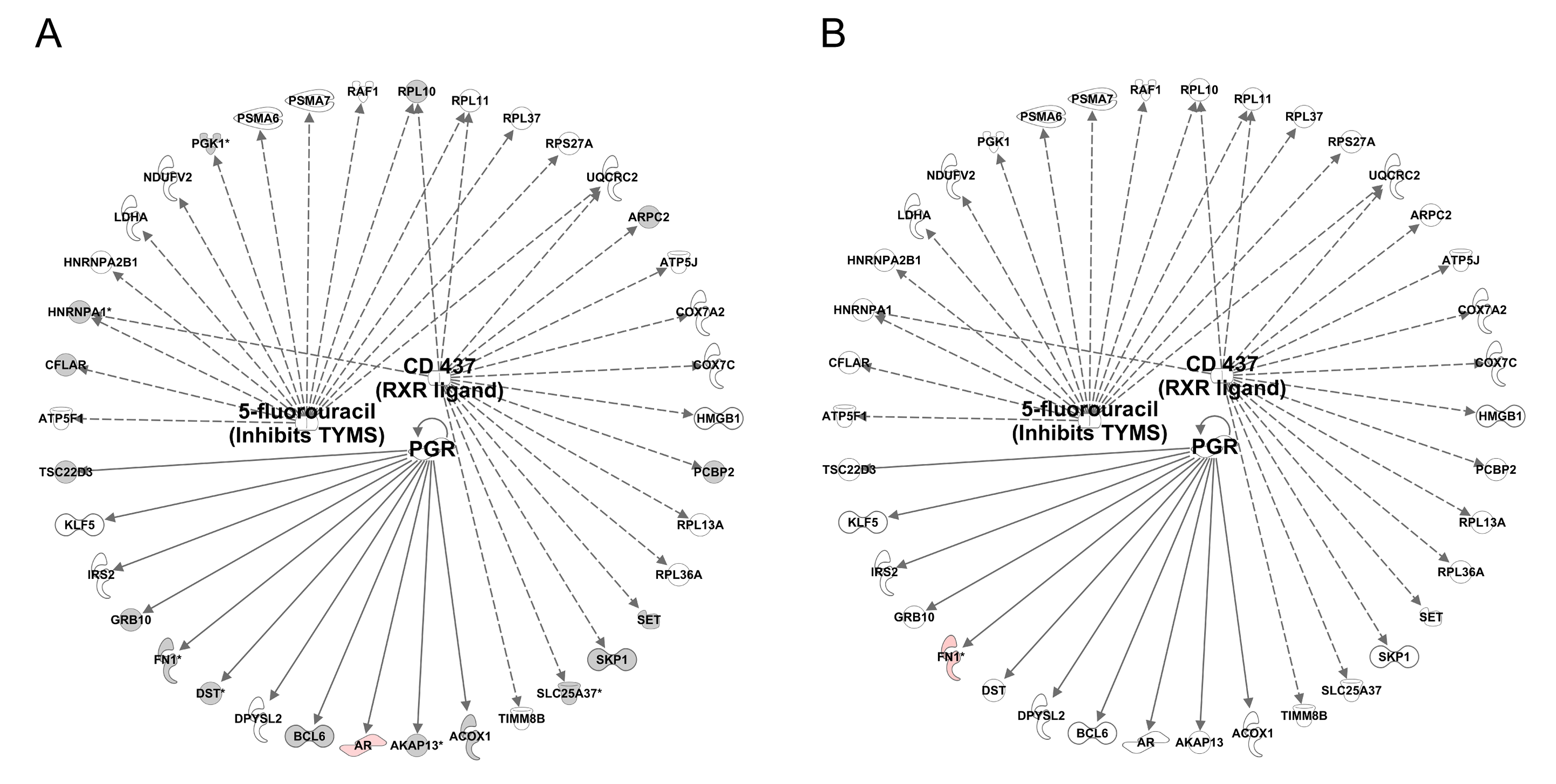

Supplement: Figure S4 — Gene networks positively associated with age in human muscle contain RNA's which are not regulated by either A) acute resistance ET (Trappe data-set GSE28422) nor B) 6 wk endurance exercise training (Keller et al 2011 JAP). (TIF) [file pgen.1003389.s009.tif]

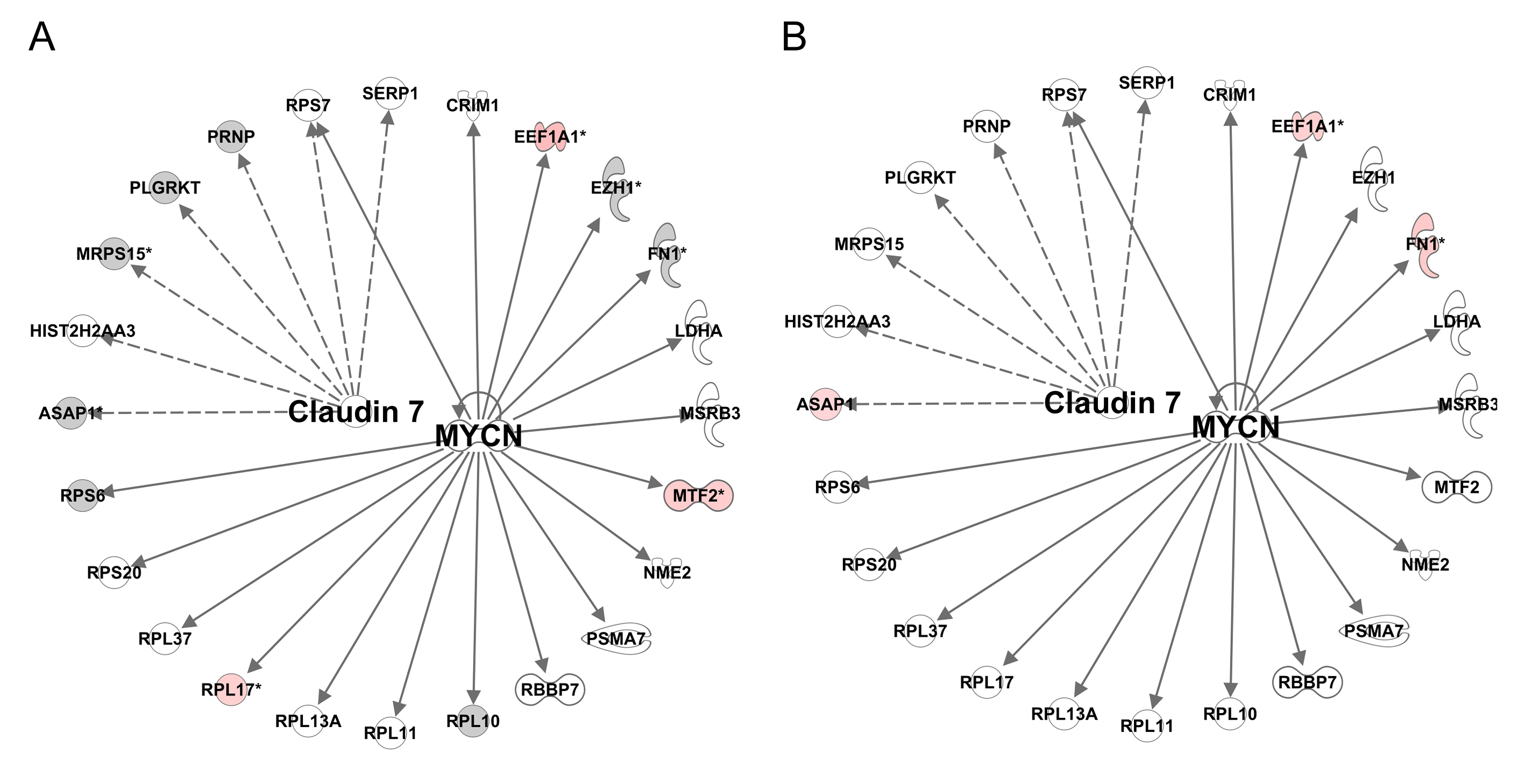

Supplement: Figure S5 — Age related gene networks where the central node is inhibited with age contain RNA's which are positively correlated with age and increased with A) acute resistance ET (Trappe data-set GSE28422) and B) 6 wk endurance exercise training (Keller et al 2011 JAP) providing a clear indication that age and exercise are not simple ‘opposites’ at the molecular level. (TIF) [file pgen.1003389.s010.tif]
